# AFM Nanotribomechanical Characterization of Thin Films for MEMS Applications

**DOI:** 10.3390/mi13010023

**Published:** 2021-12-25

**Authors:** Corina Bîrleanu, Marius Pustan, Florina Șerdean, Violeta Merie

**Affiliations:** 1 Micro-Nano Systems Laboratory, Technical University of Cluj-Napoca, 103-105 Muncii Blvd., 400641 Cluj-Napoca, Romania; Corina.Barleanu@omt.utcluj.ro (C.B.); Marius.Pustan@omt.utcluj.ro (M.P.); Violeta.Merie@stm.utcluj.ro (V.M.); 2 Mechanical Systems Engineering Department, Technical University of Cluj-Napoca, 103-105 Muncii Blvd., 400641 Cluj-Napoca, Romania; 3 Materials Science and Engineering Department, Technical University of Cluj-Napoca, 103-105 Muncii Blvd., 400641 Cluj-Napoca, Romania

**Keywords:** MEMS, thin film, AFM, stiction, friction, adhesion, Young’s modulus, hardness

## Abstract

Nanotribological studies of thin films are needed to develop a fundamental understanding of the phenomena that occur to the interface surfaces that come in contact at the micro and nanoscale and to study the interfacial phenomena that occur in microelectromechanical systems (MEMS/NEMS) and other applications. Atomic force microscopy (AFM) has been shown to be an instrument capable of investigating the nanomechanical behavior of many surfaces, including thin films. The measurements of tribo-mechanical behavior for MEMS materials are essential when it comes to designing and evaluating MEMS devices. A great deal of research has been conducted to evaluate the efficiency and reliability of different measurements methods for mechanical properties of MEMS material; nevertheless, the technologies regarding manufacturing and testing MEMS materials are not fully developed. The objectivesof this study are to focus on the review of the mechanical and tribological advantages of thin film and to highlight the experimental results of some thin films to obtain quantitative analyses, the elastic/plastic response and the nanotribological behavior. The slight fluctuation of the results for common thin-film materials is most likely due to the lack of international standardization for MEMS materials and for the methods used to measure their properties.

## 1. Introduction

Nanotribology is a branch of tribology, which involves the interactions between two relative moving materials in contact at a nanometer or atomic scale. Nanotribology has been stimulated by the manufacture of microelectromechanical systems (MEMS). With the advent of scanning force microscopy (PMS), the experimental approach to nanotribological regimes has been substantially advanced. The application range has been extended, including triboelectric nanogenerators [1], while the most common examples of nanotribological phenomena include hard disks, MEMS and nano-electro-mechanical systems (NEMS).

Tribology is a surface phenomenon that can be significantly affected by a very large surface-to-volume ratio in a micro or nanostructure. Low mass, light load, elastic deformation and slight wear or absences of wear are typical of nanotribology. It has been widely perceived that various conditions of tribological testing, such as load, speed, temperature, surface free energy, surface topography, environment, etc., play a major role in nanotribology. The experimental study of nanotribology is made possible by the use of a surface force (SFA), atomic force microscope (AFM), friction force microscope (FFM) and disk ball nanotribometer. This paper discusses various aspects of nanotribology, including nanofriction and nanosaturation for MEMS. Nanotribological measurement methodologies and mathematical relationships between frictional, bending and torsional forces based on AFM/LFM (lateral force microscopy) are comprehensively reviewed. The influences of different experimental conditions on nanotribology are described with various examples. Simulation techniques for nanotribology are also highlighted.

Evaluation of the mechanical properties of nanomaterials, in particular thin films, which are used in MEMS devices, is essential regardless of the marketable aspect of applied devices for MEMS.

Nanotechnology, known to be technology performed at the nanoscale with real-world applications [2], has determined the advancement in the field of innovative micro/nanosystems through the introduction of new and improved materials and processes and led to the accelerated evolution of MEMS/NEMS. In recent years, there has been a great number of emerging applications in this field.

Regardless of the rising popularity and of the technological growth in MEMS/NEMS applications, the severe tribological (friction and wear) problems tend to ruin their performance and efficiency. In fact, several studies have shown that the factors that limit the expected vast effect of nanotechnology on our mundane lives are the tribological and mechanical aspects of these devices [3,4,5,6,7]. Miniaturization and the subsequent development of MEMS/NEMS demand system components with better tribological efficacy and a good understanding of essential phenomena underlying wear, lubrication and friction on micro- and nanoscales [3,4,5]. The components of the micro/nanostructures are very lightweight (around a few micrograms) and are used under very small loads (from a few micrograms to a few milligrams). The switch from macro- to microscale determines an increase in the ratio between the surface area and volume, causing a severe concern from a tribological point of view. The forces that occur on a microscale surface, such as friction, adhesion, meniscus forces, viscous drag and surface tension being in direct correlation to the area, increase significantly, limiting the durability and reliability of MEMS/NEMS. Hence, at the nanoscale, friction and wear for the case of nanostructures on which light loads are applied are highly conditioned by the surface interactions.

**Figure 1:**
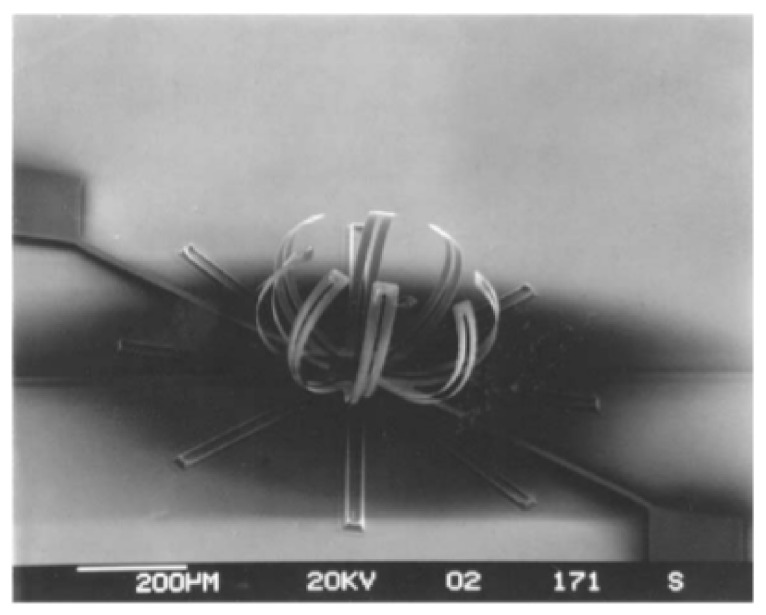
Thin film NiTi microwrapper. Reproduced with permission from Ref. [15].

**Figure 2:**
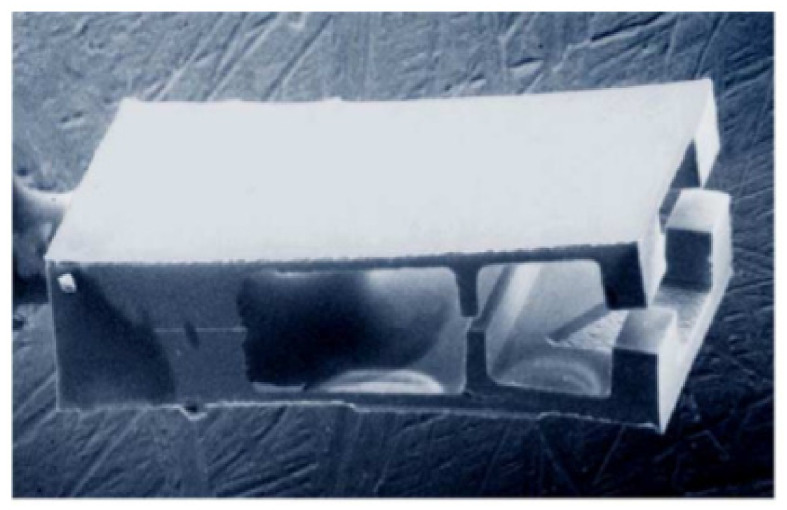
TiNi/Si microgripper with cantilever structure. Reproduced with permission from Ref. [16].

**Figure 3:**
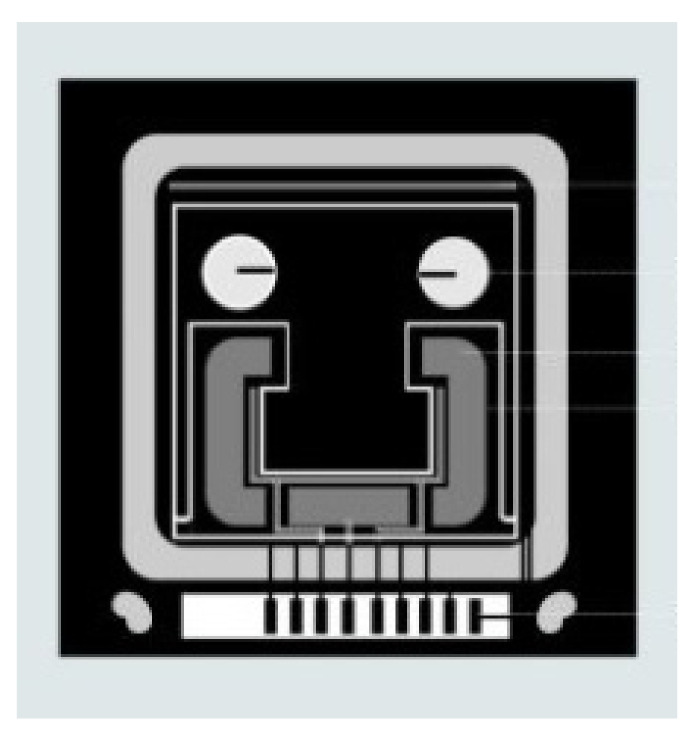
Microdevice with thin-film temperature sensor. Reproduced with permission from Ref. [20].

**Figure 4:**
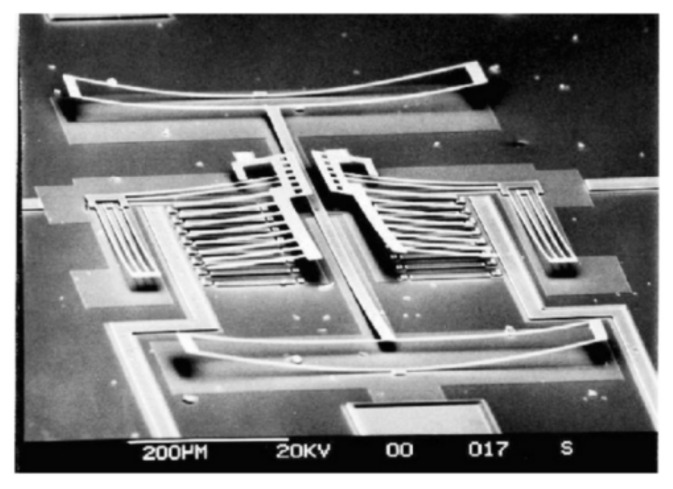
Effect of residual stress on free-standing structure. Reproduced with permission from Ref. [21].

**Figure 5:**
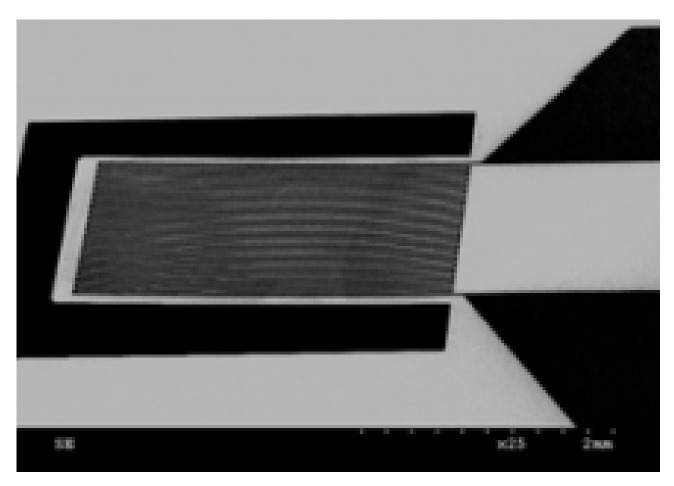
Si microcantilever structure with TiNi electrodes. Reproduced with permission from Ref. [16].

The thin-film materials are the decisive aspects of uninterrupted technological advancements made in the field of electronic, photonic and magnetic devices. When materials are processed into thin film they can be easily integrated into various types of devices [8]. The thin film coatings are used in various applications (Figure 1, Figure 2, Figure 3, Figure 4 and Figure 5) such as machining tools, microelectronics, data-storage media, biomedical prosthetic components, and surgical tools, etc. When thin coatings are complemented by some functional improvements, then an important increase in the mechanical components’ reliability can be observed. Furthermore, the resistance to mechanical deformation of the components needed during device usage can be provided by hard thin films [6,9,10,11]. Due to the improvement made by researchers in the field of nanomaterials, thin-film systems can have only a few 100′s of nm; however, they can enhance the efficiency of thin coatings used in these crucial industrial areas [9,11,12,13,14].

Another use of thin films with a large commercial potential is to protect structural materials in high-temperature environments. Further progress can be made if the multilayer coatings or the so-called continuously graded coatings are used [17]. The thin-film thickness normally includes one or few atomic layers. The mechanical properties of these films are much more strongly influenced by grain size, grain shape, crystallographic texture, the interatomic potentials and surface energy [14,17,18,19].

In thin film for microelectromechanical systems (MEMS), tribological and static interfacial forces occurring at the contact interface are comparable with the driving forces within a micro and macro scale device. In this case, micro and macroscale tribological aspects (lubrication, wear, friction aspects, etc.) are inefficient. New nanotribological methods must be used for MEMS devices of thin films with moving structures.

In this paper, we review the fundamental and critical tribological aspects related to micro- and nanoscale mechanical contacts and developments in MEMS thin-film structures. Tribo- mechanical aspects that arise from the interaction of two bodies in contact and that are in relative motion, starting from the atomic- to microscale, are required to be apprehended in order to have basic insight on adhesion, wear, friction, scratching, indentation and lubrication phenomena. Many interactions that can be overlooked at the macroscale are crucial at the microscale due to the fact that they can influence the stabilities and performances of microdevices. Thus, adhesion [7,22,23,24] micro/nanofriction [25,26,27,28], hardness [29], and Casimir effects [24,28] between micro-objects have been the subject of much research. The core of several researchers’ [24,30,31] investigations was the effects of the film thickness, process pressure and process power on the residual stress of several thin films, while Harrison et al. [25] analyzed the mechanical and tribological properties of diamond-like carbon films and self-assembled monolayers. Other researchers such as Conde et al. [32] examined the electrostatic actuation of thin-film silicon MEMS using optical and integrated piezoresistive and magnetic detection, while Fu et al. [16] approached some crucial issues when developing TiNi thin films, including preparation and characterization aspects, residual stress and adhesion, frequency improvement, fatigue and stability.

TiNi thin film is considered a basic technology for operating MEMS devices where force and high stroke are essential, as well as for intermittent operating conditions or in extreme environments such as radioactive, space, biological and corrosive conditions. TiNi film applications in MEMS are focused on microactuators, such as micropumps, microvalves, micro-grippers, springs, microspacers, micro-positioners and microrappers, etc. [27].

The introduction in 1985 of the AFM techniques provided a method for measuring ultra-small forces between a probe tip and a surface, and it has been applied for morphological and surface roughness at the nanoscale, as well as for friction and adhesion measurements. The AFM is also being exploited for different investigations, including scratching, indentation, wear, and detection of material transfer, etc. In order to gain some insight on the molecular scale operation of lubricants and to study the frictional properties, the investigated surfaces have been moved laterally. In addition to this type of research, investigations in which the AFM tip was used to simulate the contact of a single asperity with a solid or lubricated surface were performed, and they proved that the connection between friction and surface roughness is not always elementary or straightforward [3].

The main elements to consider regarding MEMS tribological aspects are friction control and wear minimization. Even if MEMS devices are tiny, they are on the order of micrometers rather than nanometers. For example, a 10μm cube of silicon has around 1013 atoms; hence, large deviations of mechanical property values from familiar values are not likely to happen. The intensive research from the last years has led to the development of several measurement methods that were later used to determine various values of material characteristics. Nonetheless, when comparing the studies on measurement methods and their usage on thin-film materials, it can be observed that there are inaccuracies in the experimental results obtained using each method [33].

As the size of the devices decreases to micro- and nanoscales, the surface-to-volume ratio increases and the effects of body forces (gravity and inertia) become insignificant compared to those of surface forces (van der Waals, capillaries, electrostatic chemistry and adhesion). In microelectromechanical systems (MEMS), the tribological and static interface forces are comparable to the forces that lead to the movement of the device. In this situation, macroscale lubrication and wear attenuation methods, such as the use of bulk fluids and micrometric thickness coatings, are ineffective; new nano-engineering approaches need to be used for MEMS devices. We review the fundamental tribological issues related to MEMS mechanical contacts made from thin films.

In this paper, we present on the one hand a review of the important aspects of nanotribological, nanomechanical and material of thin films and, on the other hand, a characterization of thin films conducted using AFM with nanoindentation modules. Studies were conducted on a few different mono- and multilayer thin-film materials. This paper also encompasses a review of micro/nanoscale adhesion, friction, and wear studies of materials for MEMS/NEMS and component-level studies of stiction phenomena in MEMS/NEMS devices.

The paper is an excellent reference for both academic and industrial researchers working in the fields of nanotechnology, tribology, mechanical engineering, materials science and engineering, MEMS, NEMS, and biomedical devices. It will also be of interest to those pursuing AFM microscopy, nanoimaging, sensors, actuators, and aerospace, etc. This work provides an assessment of the current state-of-the-art of nanotribology within the context of MEMS, nanotechnology and microsystems.

## 2. AFM Measurement Technique for Tribological Characterization of Thin Film Materials

Because the dimensions of MEMS are the order of micro and nano, the measurement of mechanical properties is difficult to perform due to aspects such as accuracy, repeatability and reliability of measurements. In general, the microdevices are made by deposition and etching processes; thus, the specimen has to be produced by the same procedures used in the device manufacturing. In these conditions, the possible investigative techniques include optical or scanning electron microscopy, interferometry, mechanical or optical profilometry. In this paper, we used the force/displacement that results in deformation for measuring the mechanical properties of thin-film samples for MEMS. After applying the force and measuring the displacement, elastic, inelastic or strength properties can be determined from the obtained model [34,35]. Nonetheless, the usage of direct methods for obtaining the mechanical properties is not appropriate in most cases when MEMS materials are in question.

### 2.1. Basic Topographic Imaging

Atomic force microscopy is one of the most adaptable and capable microscopy technologies when it comes to studying samples at the nanoscale. The adaptability is proven by the fact that an AFM can not only determine the three-dimensional topography, but it also allows several types of surface measurements that can be used by scientists and engineers. The capability is proven by the fact that an AFM can generate images at atomic resolution with angstrom scale resolution height information, even if there is a minimum sample preparation.

The AFM technique (including lateral force module and nanoindenter) has been widely preferred by scientists when trying to determine the mechanical characteristics of small volumes of materials, such as thin films [36]. Many applications require, besides determining the experimental values of the elastic modulus and hardness of thin films, the interpretation of the nanotribological effects, such as the deformation behavior of the film due to lateral movement of sharp contacts. The two important aspects that should characterize most hard thin-film coatings are a low coefficient of friction and a high strength adhesive bonding with the underlying substrate. Sometimes, considering the application, a low wear rate under cyclic scratching may also present interest, but usually it is not a crucial aspect for a thin film with a really high hardness. The optimal instrument to investigate the tribological characteristics of such thin-film materials at nanolength scales is AFM with lateral force measurement capability.

The oldest and the most basic imaging mode of the AFM is the contact mode atomic force microscopy (CM-AFM), which consists of bringing the tip into direct contact with the sample surface, and as a consequence, the cantilever is deflected (repulsive force) [36]. When actually using them, most AFMs work with the beam deflection method, and thus, the control signal comes from the photodiode, and it corresponds to the cantilever’s vertical deflection. The feedback setpoint value that provides the values at which the images are taken can be elected by the operator considering the experiment conditions. During the scanning of the surface performed by the tip, the normal signal from the photodiode is maintained equal to the setpoint by automatically adjusting the Z-scanner.

### 2.2. Mapping of the Frictional Force: Lateral Force Microscopy (LFM)

Understanding information regarding the nanomechanical properties of the samples, including material stiffness (moduli), friction coefficient, viscosity, hardness, and elastic-plastic yield points, can be provided by lateral force microscopy. In addition to other uses, LFM has been chosen to map the spatial variations in the surface characteristics of heterogeneous polymers [37,38], thin films [39] or surfaces mapped using lithography methods [38,40]. Bhushan et al. [38] proved the application of LFM in the case of analyzing the error for arrays of micromirrors. Kim et al. [38,41] applied LFM to determine the forces demanded for the collapse of photoresist patterns. Thus, along with plain imaging of lateral forces, this type of microscopy can also be used for the mechanical testing at the nanoscale of thin films and/or small structures [38]. It is worth mentioning that the forces determined by LFM can vary between piconewton and micronewton values, with respect to the application considered. When trying to estimate the force ranges, Amontons’ law [38,42] is considered to be valid, namely that the friction forces are proportional to the normal load.

The basis of LFM for the XE-series is very much similar to that of the contact mode AFM. Although in contact mode the cantilever deflection is measured in the vertical direction in order to collect data on the sample surface, in LFM, the cantilever deflection is measured in the horizontal direction. After applying a force to the cantilever when it advances horizontally over the sample surface, a lateral deflection of the cantilever occurs, and its magnitude is established based on several factors such as sample surface topography, frictional coefficient, lateral spring constant of the cantilever and cantilever movement direction. LFM for the XE-series is very convenient when analyzing samples whose surfaces are made of inhomogeneous compounds. Another use is enhancing the contrast at the boundary of a suddenly modified slope on the surface of the sample or at the margin between distinct compounds. Data on surface friction and topography are obtained from the LFM results. Thus, when analyzing the values obtained from the LFM measurements, it is imperative to identify which results are due to the difference in the values of the frictional coefficient and which are due to the change in the surface topography of the sample. This can be achieved with a correlation to the topography image.

### 2.3. Nanoindentation

Nanoindentation is one of the most used techniques for determining mechanical properties at small scales. When performing a nanoindentation test, distinct phenomena can be detected due to the high-resolution load-displacement data, such as dislocation source activation, phase transformations and shear instability initiation. More quantitative details regarding these occurrences can be obtained due to the improved techniques, such as high-temperature nanoindentation testing or indented volume in situ imaging. They also open doors for future scientific analysis [43,44].

Due to the advancements in the last decades in the field of sensors and actuators, which allows testing to take place at submicron scales, a drastic change has resulted in the way indentations are performed. The technique introduced as a consequence, named nanoindentation, is now almost omnipresent when it comes to determining the mechanical properties of different surfaces. One of the most important areas where nanoindentation is applied is elastic modulus and hardness measurements [9,45]. However, other mechanical parameters such as hardening exponents [46], creep parameters [47,48,49], and residual stresses [31,50,51] have started to be investigated, presenting opportunities for solving applications from physical sciences. Another use of nanoindentation for property extraction has been recently presented in several reviews [44,52,53,54].

Nanoindentation is one of the XE Modes that determines the hardness of a local region. A sharp tip is lowered onto a sample and a small indent is formed. The value of the hardness is correlated to the area and depth of the indent. The depth of the penetrations is in the nanometer range, but the results can be accurately determined by automatically performing multiple tests. Following this procedure, Young’s modulus and the strength of different thin films can be determined. Based on the data obtained from the indentation, other properties such as adhesion, elasticity, adhesion, tribology and creep can also be computed.

**Figure 6:**
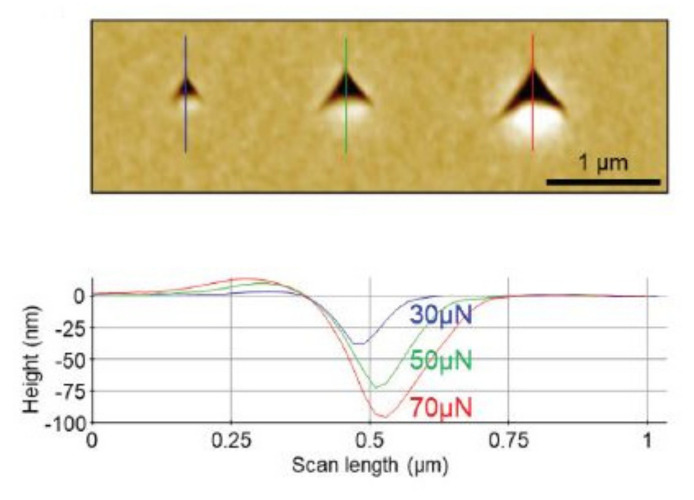
Height image obtained after performing nanoindentation experiments with three different force setpoints using the AFM indentation tip.

The procedure of indentation is not complicated at all. The difficult part comes with the analysis. The calibration of the force and the displacement (depth) are crucial aspects to be considered. The total force that acts on a sample is influenced by factors such as the tip shape, the cantilever force constant, and some tip mechanical properties. A Berkovich indenter is a single-crystal natural diamond tip with a three-sided pyramidal shape that is frequently used. It is set up on the beam so that a parallelism occurs between one sharp edge and the long axis of the beam. The indenter presses down for a couple of seconds onto the sample surface at a given force. The geometry of the indentation mark can be analyzed using a high-precision atomic force microscope, as presented in Figure 6. It is worth mentioning that the AFM is the only technique efficient enough to obtain the exact geometry of small areas.

**Figure 7:**
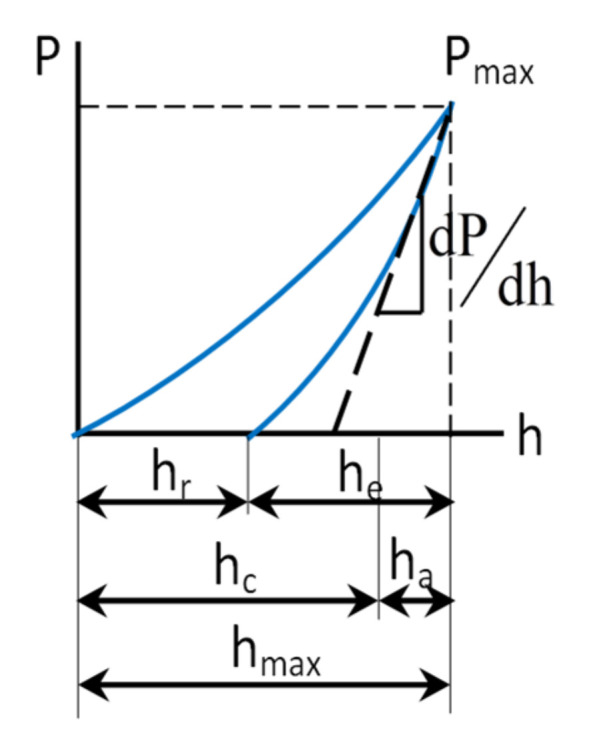
A loading and unloading displacement curves during nanoindentation.

The result of the division between the loading force and the indentation projected residual area is the value of the hardness. Based on the slope of the unloading curve, the value of Young’s modulus of elasticity can be determined. In Figure 7, typical loading and unloading displacement curves are presented. They are obtained from a nanoindentation test with a maximum load of P_max_ and a depth beneath the sample-free surface of h_max_. The sample modulus and hardness are computed based on the contact circle depth h_c_ and the elastic unloading curve slope. In the same figure, the residual impression depth h_r_ and the displacement related to the elastic recovery during unloading h_e_ are also presented. The loading and unloading curves would look alike in the case of compliant materials because the deformation is for the most part elastic.

**Figure 8:**
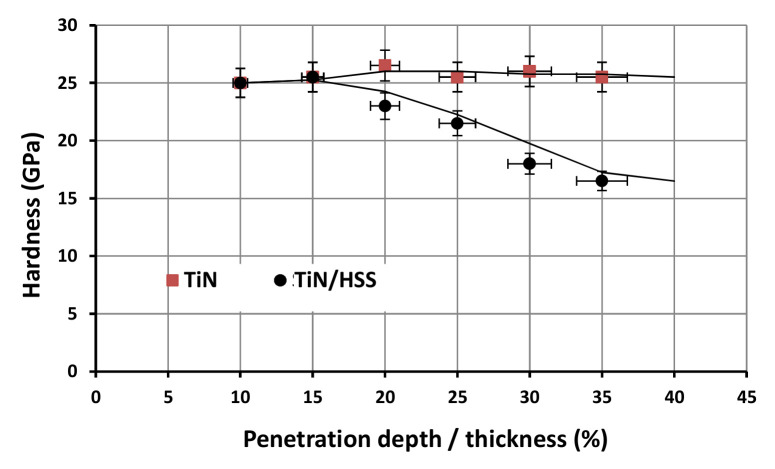
Comparison the hardness between bulk TiN and TiN/HCC as a function of the ratio d/t.

Lichinchi et al. [55] analyzed the impact of the substrate on the dispersion of plastic deformation considering the penetration depth in the case of coated systems. When measuring the hardness, to avoid substrate effects, the depth of penetration was chosen to be less than 10–20% of the film thickness. The authors made a comparison between a bulk TiN sample and an HSS substrate with a 2 µm thick TiN coating. In Figure 8, the hardness versus the ratio d/t is presented, and it can be observed that the hardness of the coated system decreases up to maximum deviation of 33% when compared to the bulk TiN.

Jung et al. [56] notified that for the oxide film, they expected similarity of the properties with those of bulk amorphous silica, SiO_2_. The values that are reported for this material in the open literature are around the values of E = 70 GPa and H = 10 GPa. They are comparable to E_f_ = 72.5 GPa and H_f_ = 11.5 GPa obtained for their oxide film. It is harder to compare data for the nitride films with values for bulk silicon nitride because the data available in the open literature is for polycrystalline material and it depends on a high degree on the microstructure (crystalline phase, grain size, and additives, etc.).

**Figure 9:**
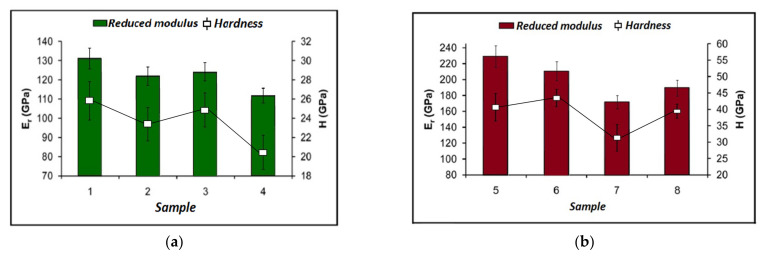
(**a**) Plots of E_r_ and H data from 45 μN nanoindentation tests on 50 nm TiN thin-film samples; (**b**) data from 60 μN nanoindentation tests on 150 nm TiN thin-film samples.

For the TiN thin film, a full nanomechanical characterization was conducted for the TI 950 from Hysitron. The samples consist of 50 and 150 nm TiN thin films deposited on quartz glass substrates using several sputtering techniques. In general, the testing of the thin films is performed only on the top 10–20% of the thickness of the film so that the influence of the substrate on the measurements is avoided. Each sample was indented eight times with maximum indentation loads of 45 μN for the thinner films and of 60 μN for the thicker ones. The loading lasted five seconds, then it was followed by a two-second hold in order to obtain the indent, and in the end, the unloading lasted five seconds. The results obtained for the mechanical properties are presented in Figure 9a,b. The measurements were conducted for the two properties with depth profiling nanoindentation. The results indicate a logarithmically decreasing trend with respect to the contact depth of the indenter for all samples. More testing modes were used, but the obtained results are in excellent agreement indicating the accuracy of the obtained results. When trying to remove the effect of a hard film deposited on a soft substrate and obtain real, quantitative values for the mechanical properties of the film surface (1–2 nm penetration depth) Modulus Mapping™ was used.

**Figure 10:**
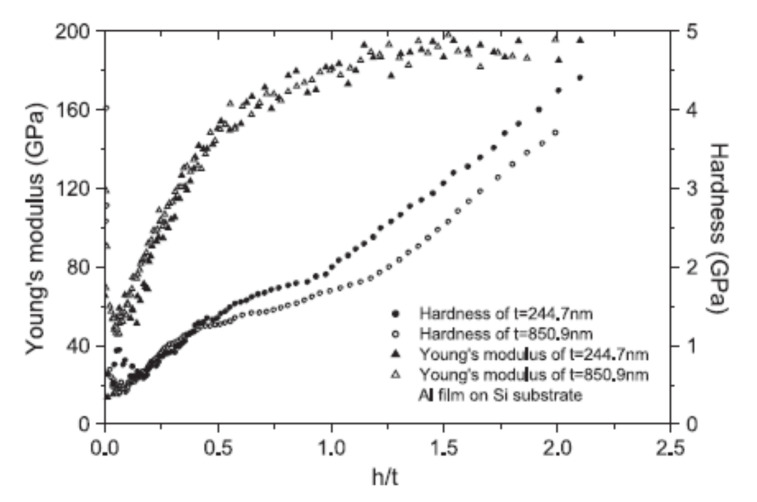
Hardness and Young’s modulus versus normalized indentation depth. Reproduced with permission from Ref. [57].

The composite hardness and modulus of elasticity for Al–silicon systems with different thicknesses of the thin films are presented in Figure 10 according to [57]. As it can be seen, the composite hardness for two Al–silicon systems with film thicknesses of 244.7 and 850.9 nm, respectively, are shown with respect to the ratio h/t (normalized indentation depth). The results for the Al film with a thickness of 52.3 nm were eliminated because the hardness fluctuated. Furthermore, the investigation focused on determining the effect of the film thickness (when deposited on the same substrate) on the experimentally determined composite hardness and modulus of elasticity. The experimental results indicate an increase in the film hardness with a decrease in the film thickness, while the values of the modulus of elasticity are not affected by the film thickness. Due to the fact that the contact areas were determined using the Oliver and Pharr method, constant values were obtained for the hardness only in a tiny area while the depth of the indentation was less than the thickness of the film [57,58,59].

Volinsky et al. [60] used an approach consisting of heating the local area and using a drift correction up to 130 °C in order to prove the utility of high-temperature nanoindentation experimental tests. As the technology progresses and more heating devices become accessible to researchers, nanoindentation at higher temperatures may be achievable. A major aspect to be considered when designing such a device is the cooling system for the sample as well as for the indenter tip, to guarantee the least possible thermal drift. Larger temperatures influence the yield stress of the film by activating the plastic deformation process. At a small indentation depth, the increase in temperature up to 130 °C determines a 60% drop in hardness values. For indentation depths larger than 500 nm, the influence of the temperature drops because of the substrate hardness.

Verdyan et al. [61] performed studies using the AFM on the morphology and the local mechanical properties of gold nitride thin films. The investigated properties are the hardness, modulus of elasticity, and scratch resistance of the film. The techniques used to obtain the experimental results were nanoindentation and nano scratch tests. They installed a diamond tip on the metal foil cantilever of the AFM used. The performed experimental test indicated values of 2–3 GPa for the hardness and a value close to 100 GPa for Young’s modulus. The measurements were processed according to Oliver and Phar’s model, and the obtained data were interpreted with respect to dislocation pinning, boundaries and grain size. A vast area of applicability for gold films is electric low-resistance contacts in the microelectronic industry and, thus, the increased commitment to study gold nitride thin films. Nevertheless, since gold thin films have a hardness around 2 GPa or even less, they are not used to increase the coating hardness. Instead, for this application, metals such as Fe, Co, and Ni, or Ti, Pb, and As are frequently used [61].

**Figure 11:**
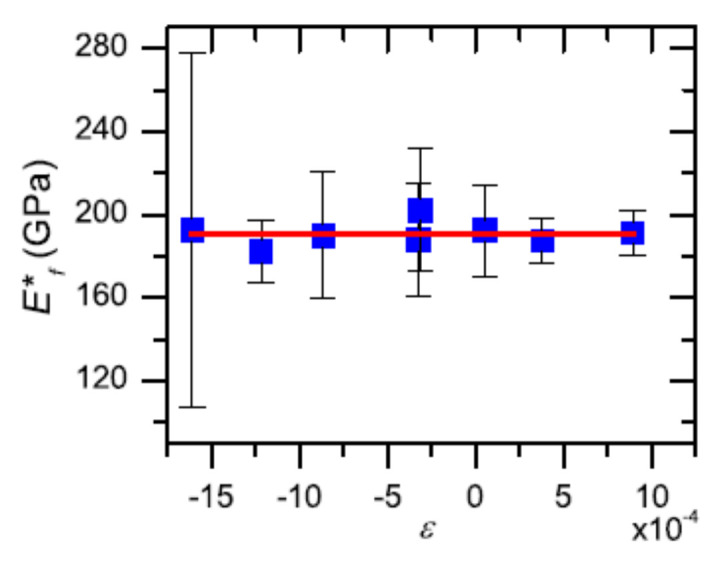
A–SiNxHy thin film E*_f_ vs. ε calculated using A_AFM_. The solid line in each plot denotes the average value of the data [62].

Soh et al. [62] used nanoindentation to investigate a–SiNxHy thin films of variable ε between −1.6 × 10 − 3 and 0.9 × 10 − 3 H and $E_{f}^{*}$, calculated using the multi-point unload technique, were observed to be dependent on ε. However, AFM imaging of the residual impressions on the thin-film surface indicates some degree of elastic recovery. The degree of elastic recovery was found to be dependent on ε, with thin film A_AFM_/A_(hp)_ monotonically increasing with ε. $E_{f}^{*}$ calculated using A_AFM_ was independent of ε, and approximately 190 GPa (Figure 11). Since strain is present in most thin films and multilayers, often due thermal mismatch, imaging of residual impressions is necessary in order to measure the actual contact area and, thus, accurately determine the elastic modulus of thin films using nanoindentation.

**Figure 12:**
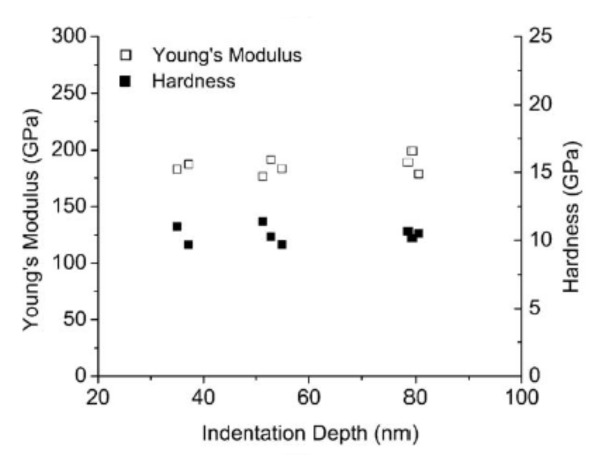
Hardness and Young’s modulus vs. indentation depth. Reproduced with permission from Ref. [63].

Cao et al. in [63] investigated experimentally the influence of the microstructure/thickness of the thin film on the deformation occurring due to contact in polycrystalline Au and Ag thin films obtained by electron-beam deposition on silicon substrates. The deposition on Si substrates was conducted for more thin films with various thicknesses. First, the mechanical properties of the substrate were determined regardless of the indentation depth, and the obtained results are encompassed in Figure 12. As it can be seen, if the average values are computed for Young’s modulus and hardness, the obtained values are in good accordance with the values reported in the scientific literature. Then, based on these values and using the modified King’s model, the properties of the thin films were determined.

**Figure 13:**
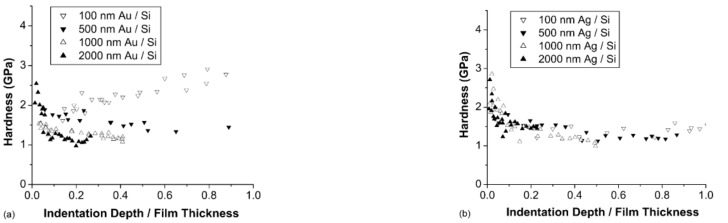
Hardness vs. normalized indentation depth for Au films in (**a**) and Ag films in (**b**). Reproduced with permission from Ref. [63].

The results obtained for the hardness of Au and Ag films of different thicknesses (with respect to the normalized indentation depth) are shown in Figure 13. As it can be observed, the hardness increases with the increase in the normalized indentation depth in the case of the thin film with a thickness of 100 nm due to the effects of the substrate modulus mismatch. The indentation depth has a visible influence for normalized indentation depths of up to ~0.2 in the case of the thicker films because of geometrically necessary dislocations at small scales. This value of 0.2 is in good agreement with the critical ratio of 0.18–0.22 determined experimentally for the Au films on Ni substrates.

## 3. Thin Films for MEMS

A broad variety of materials have been investigated. Among these are materials typically used in the MEMS applications. Among these are materials typically used in the MEMS applications. MEMS/NEMS devices are traditionally made of silicon, which have poor tribological properties. To improve the tribological performance of silicon, the researchers investigated various thin films/coatings that include diamond-like carbon coatings (DLC), self-assembled monolayers (SAM), polymers, perfluoropolyether films and different thin films. The nano/micro-tribological properties of these materials are governed by several parameters, such as their physical structure, chemical composition, surface properties, including surface energy/wettability and resistance to interfacial shear, and mechanical properties, such as elastic modulus influencing contact area. A brief presentation of the nano/micro-tribological properties of the adhesion, friction and wear durability of these MEMS/NEMS materials are presented in this paper.

The largely used materials in MEMS/NEMS are single-crystal silicon and silicon-based materials. The disadvantage of silicon-based MEMS/NEMS devices is their mechanical and electrical behavior at high temperatures. Lately, the focus of researchers for applications involving micro- or nanosensors/actuators used at high temperatures has been on SiC [64,65]. Another disadvantage of silicon-based materials is that they break easily. According to recent research, silicon has inferior fatigue properties [5,9]. A material that presents interest is gold due to its high conductivity. There has been a wide range of applications for electroplated gold films [4].

Silicon dioxide is one of the most used materials for making MEMS. In micro-machines surface coatings, SiO_2_ is used as sacrificial material as it is easy to dissolve and remove without having to attack the thinner polysilicon layer. Other thin films that have presented interest to researchers lately are TiNi thin films due to their extraordinary properties. Micro-actuators that incorporate such thin films have been used more and more in the quickly expanding area of MEMS [16]. Based on metals such as Ag, Al, Au, Co, Cu, Cr, Mn, Ti, Zr or nonmetals such as Ge, Si, ZnS, SiO, Te, many structures of thin films with different compositions have been developed. However, the characterization of such thin films from the mechanical point of view is still needed, at least at the nanoscale. It was already seen that one of the most common realizations of MEMS processes involves depositing a thin layer on a base coat. These layers, of course, are called thin films.

**Figure 14:**
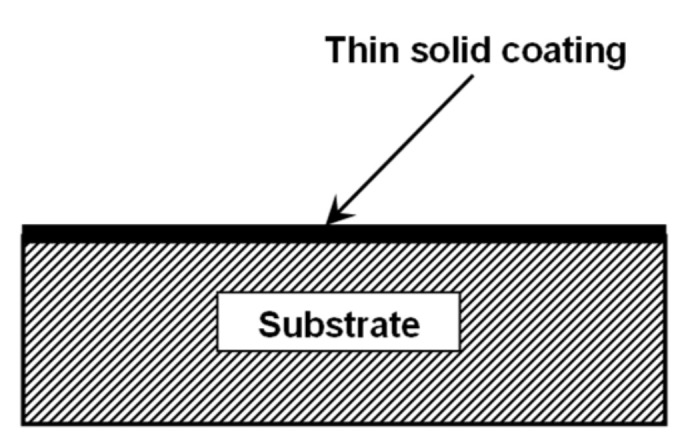
A typical system of thin, solid coating/substrate.

Many different kinds of thin films are used in the fabrication of MEMS. In general, the efficiency of a mechanical system is influenced by factors such as interfacial behavior of/between its individual components and surface properties. The last-mentioned factor is extremely important in the actualization of designed functions when it comes to micro/nanotribology applications. When certain properties are desired, techniques that deposit thin layers on different substrates (thin solid coatings) are chosen. Obviously, the layer materials are different from the substrate and the layer thickness is usually in the nanometer to micrometer range. In Figure 14, a typical system of thin solid coating on a different substrate is presented. The required properties for a certain application (for example, low friction or corrosion resistance or wear resistance) can be achieved by choosing wisely the materials for the layer coating as well as the method of deposition. Thin solid coatings have been of great interest to researchers due to their vast area of applicability, and the research conducted in this field has been focused both on the choice of coating materials as well as on different preparation techniques [66,67].

Due to the thin film deposition process, residual stresses often occur (compressive or tensile) in thin films. Sometimes the residual stress is an advantage for the MEMS producers; other times, however, tensions arising can cause critical issues in a MEMS device. Consequently, comprehending the mechanical properties of thin films becomes mandatory. Lately, due to the intensive research in this field, novel thin coating materials were developed that captivated the attention of researchers.

**Figure 15:**
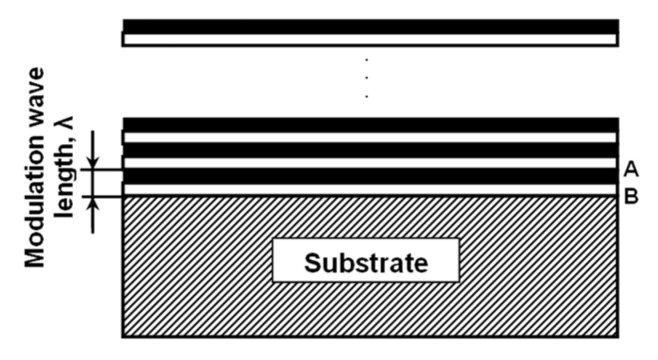
Schematic of the structure of superlattice film.

In 1970, Koehler [68] focused his research on designing strong solids by using the method of alternating layers of crystals A and B, as presented in Figure 15. The name given to these multilayer materials is heterostructures or superlattices. In order to have the desired outcome, the two alternating layers should be chosen so that they are characterized by similar lattice parameters and thermal expansions but different elastic constants. Assuming that E_A_ > E_B_, when stress is applied on the material, small displacements would occur in B layers and would advance towards the A/B interface. The displacement would be stopped from extending beyond the interface due to the repulsing force induced by the elastic strain in the A layer. Therefore, the superlattice strength is increased because only a substantial stress will be able to move material displacements from B into A. More recently, Koehler’s prediction was corroborated by Lehoczky [69], who deposited Al/Cu and Al/Ag laminate systems.

When the thickness of the layer is between 20 and 70 nm, the obtained coating is strengthened about 4.2 times than that anticipated based on the rule given by Equation (1), even if the layers of the laminate systems are polycrystalline [66]:

(1) $$H\left(A_{x}B_{x}\right)=\frac{\left[x\cdot H(A)+y\cdot H(B)\right]}{\left(x+y\right)}$$

where H stands for hardness, and x and y stand for the volume fraction of A and B, respectively.

Multilayer systems, such as metal/metal heterostructures, metal nitride/metal multilayers, metal nitride/metal nitride heterostructures, metal nitride/CNx superlattices, and metal carbide/metal superlattices, have been the focus of the work of several researchers. If the modulation wavelength was decreased to 5–7 nm, research shows that, in most cases, the strength of the superlattices was enhanced [66].

The method proposed by Koehler requires an atomic sharpening of the A/B interfaces to obtain the improved strength. The deposition conditions for such multilayer films must include a low temperature so that layer interdiffusion is avoided. Therefore, among the proper techniques to obtain such multilayer films are the PVD techniques, including evaporation and sputtering deposition.

The deposition rate can vary between 1 nm/min and several microns per minute. The process provides a structural and morphological control of the deposited films. Thin films used in this phase were deposited on two different substrates: silicon and silicon dioxide. The silicon wafers used had the following characteristics: p-type, orientation <111>, resistivity between 1 and 5 Ω∙cm and thickness in the range 475–525 μm. SiO_2_ material with a thickness of 90 nm was obtained by thermal oxidation of silicon wafers <100> n-type, thickness of 381 ± 25 mm, and resistivity between 1 and 10 Ω∙cm.

**Figure 16:**
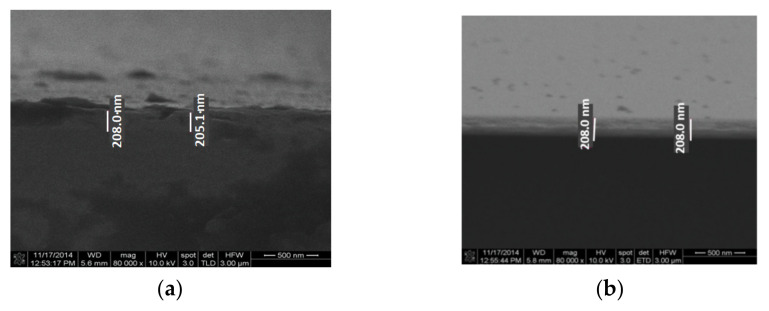
SEM investigations of metal films (**a**) Si/Al; (**b**) Si/Au.

**Figure 17:**
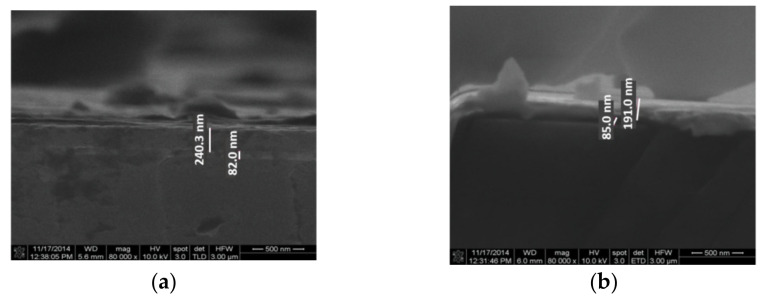
SEM investigations of metal films (**a**) Si/SiO_2_/Al; (**b**) Si/SiO_2_/Au.

The substrate has a major influence on the tribological parameters, and this is the main reason for which we have used both silicon and silicon oxide. Both wafers mentioned above have been used for depositing metal films. Aluminum and gold with a thickness of 200 nm have been deposited by the electron beam evaporation technique on both substrates (Figure 16 and Figure 17).

It is worth mentioning that the gold film was deposited without the adhesion layer (Cr or Ti) commonly used in order to not influence tribological measurements. Comparisons between parameters obtained with and without an adhesion layer will be made later.

**Figure 18:**
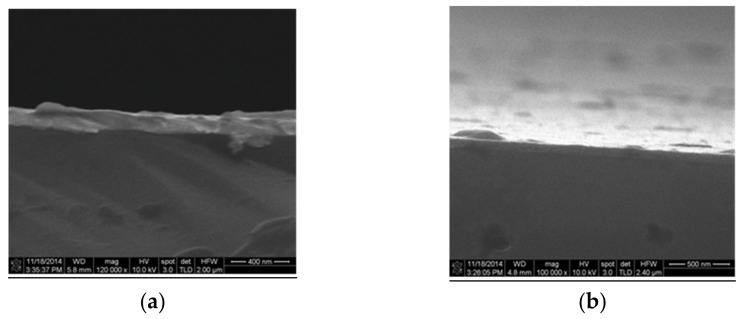
SEM investigations of metal films (**a**) Si/Ti/Au; (**b**) Si/Ni.

Another series of metals, including Ti/Au (10 nm/100 nm), Ti (100 nm), Ni (45 nm), Pt (100 nm) and Cr (150 nm), were deposited only on the silicon substrate (Figure 18).

A treatment applied to the substrate of the Si/SiO_2_ samples was thermal oxidation with the purpose of increasing the 90 nm thin film. The reaction between dichlorosilane (SiCl_2_H_2_) and ammonia (NH_3_) led to the silicon nitride (Si_3_N_4_) that was used to deposit the Si/Si_3_N_4_ samples. Using the technique of thermal wet oxidation, a thick silicon oxide (with 1.7µm thickness) was grown for the Si/SiO_2_/PolySi samples. Finally, a polysilicon layer resulted from using the LPCVD technique was deposited from silane (SiH_4_) decomposition. Two different temperatures (580 and 630 °C) were used in the LPCVD deposition technique. The measurements for the polysilicon films mechanical and tribological properties were performed for two different film thicknesses.

As a MEMS-industry innovator and leader, Micralyne’s expertise in thin films opens the door to the manufacturing of several devices, including resistors, waveguides and solder using different material thin film layers. There is a variation in thickness of thin films, which can be between a few hundred angstroms and tens of microns and a variation in materials that are deposited (Cu, Au, Pt, W, Cr, Mo, Ti, NiCu, Nb, Pt, SiO_2_, Sn, etc.).

In order to obtain the relations between the surface pattern/surface chemistry and the nanotribological properties, comprehensive nano friction and adhesive behaviors of the films with various micro-grooves were evaluated by a noise- and vibration-isolated and environment-controlled AFM in the contact mode.

## 4. Thin Film Nano-Mechanical Properties

Determining the mechanical properties of electrical materials used in MEMS devices is necessary for full exploitation of MEMS technology as well as ensuring the reliability aspect. A satisfactory comprehension of the relation existing between the processing and material properties is the key factor in manufacturing reliable MEMS. When knowing the applied force and trying to estimate the deflection, it is important to know the elastic properties of MEMS materials. For ductile material, when the MEMS deformed structure does not return to its initial state, the inelastic material properties are important.

Determining the mechanical properties of the thin film is crucial for designing MEMS devices, since the mentioned properties directly influence the following aspects:

Device performance: The performance of MEMS devices is directly influenced by the mechanical properties. In order to obtain the maximum efficiency, one requires precise values for the mechanical properties.

Reliability: The small size of MEMS devices makes them suitable to be used in unfavorable environments. In order to obtain reliable devices, their mechanical properties have to be suitable for the application where they are used.

Moreover, the need for a properties database is more and more obvious as more advances are made in this field. There is an acute need for reliable design and manufacturing information due the integration in the field of MEMS of techniques such as the rapid prototyping and also of CAD/CAE software dedicated to MEMS devices.

The elastic characteristics of micro- and nanoscale materials have been the subject of several research projects that encompassed atomistic or molecular dynamic simulations, theoretical analyses and experiments. Several experimental techniques, such as nanoindentation [70,71,72,73], have been developed in order to determine the Young’s modulus for thin films.

The basic elastic properties that govern the mechanical behavior of the thin film are the Young’s modulus (E) and Poisson ration (ν). AFM loading and unloading displacement curves are enabled to measure E together with hardness. The relation between Young’s modulus, Poisson’s ratio and the shear modulus G is:

(2) $$G=\frac{E}{2\left(1+\nu \right)}$$

Experimental tests have shown that the values of the hardness correlate with the microscale, but the values for Young’s modulus do not depend on the characteristic scale if it is larger than 200 or 500 nm. If the characteristic scale is smaller than 200 nm, the elastic constants become size dependent. Experimentally, it has been shown that the Young’s modulus and Poisson’s ratios increase if there is a decrease in the material characteristic dimension [72]. Other studies indicate that the opposite trend is true [70,73]. Likewise, the theoretical investigations arrive at the same conflicting conclusions.

Krivtsov and Morozov’s work [74] was focused on the theoretical dependency on the size of the elastic moduli of a nanocrystal. Both studied parameters are influenced by the decrease in the single-crystal strip thickness; however, Poisson’s ratio decreases while Young’s modulus increases. The conducted research further shows that the elastic moduli can vary from their values at the macroscopic scale by a factor of 2 in the case of a very thin crystal film of two atomic layers thick.

Liang et al. [28] developed a model for the dependency of the elastic moduli of Cu and Au thin films. The authors also estimated the improvement of the studied parameters. Yang [75] and Streitz et al. [76] focused on some factors that influence the nanomaterials’ elastic properties such as surface stress or surface energy. Their findings were similar to those of Krivtsov and Morozov [74]. Other researchers, Sun and Zhang [77], developed a semi continuum model for materials with nanostructures and reached the conclusion that Young’s modulus for nanoplates is around two-thirds of the equivalent bulk value. Van Workum and de Pablo [78] reached the same outcome in a collateral study that encompassed the Lennard–Jones potential. Nan et al. [79] and Sharma and Ganti [80] investigated the relation between nanocrystalline materials’ elastic properties and grain size. They determined lower values for the nanocrystalline materials elastic modulus. Lower values than those of large-scale materials were also obtained by Villain et al. [81] and Broughton and Meli [82] by using direct atomistic simulation. Additionally, Villain et al. [81] included the surface effect on the elastic properties of the studied materials. Other researchers, such as Miller and Shenoy [83], developed a basic model in which the prediction of the size-dependent elastic properties of nanoscale beams and plates was performed considering the surface tension. They arrived at the conclusion that there is a dependency between values of surface elastic constants and the effective stiffness.

Fedorchenko A.I et al. [84] have proven that there is a variation in the values of the elastic modulus with respect to film thickness. They have identified a scaling behavior and the existence of a threshold value of the film thickness h_b_ at which the influence of the thin-film surface energy becomes visible. There is an inverse proportionality between the threshold h_b_ and the bulk modulus of elasticity and also a strong dependency on the in plain strain $\varepsilon_{0}^{}$ as $\varepsilon_{0}^{-2}$. The relation that expresses the dependency between the dimensionless elastic modulus $\psi =\frac{E}{E_{\mathrm{bulk}}}$ and the dimensionless film thickness $\eta =\frac{h}{h_{b}}$ in the case of Si nanofilms can be expressed as $\psi =\eta^{0.226}$. This research proves the impact of dimensionality on the thin films’ basic parameters, with further extensive implications in developing electronic devices, such as Si-based strained nanodevices.

J.K. Luo et al. have investigated the influence of the temperature and plating current density on the modulus of elasticity for Ni thin films obtained by electroplating. A value of 205 GPa (similar to bulk Ni) was obtained for the Young’s modulus when the plating temperature was 20 °C and the low current density was J = 2 mA/cm^2^. If the plating temperature is increased to 80 °C or if the current density is raised to J = 30 mA/cm^2^ there is a drastic decrease (more than twice) in the modulus value. It is assumed that this decrease is due to the increase in coating porosity. The variation of Young’s modulus with respect to the plating conditions is of high importance when designing and manufacturing MEMS devices based on plating technologies such as LIGA [85]. The average values of the modulus of elasticity obtained with more test methods had values between 135 and 219 for SCS/SDB and between 155 and 183 GPa for SCS/Epi. For polysilicon, the average values were between 134 and 173 GPa, which were close to the values of the theoretical modulus. For the SCS samples whose tensile axis was in the (110) direction, the theoretical value for the Young’s modulus was 168.9 GPa. The same value was obtained for the polysilicon film which was (111) oriented and for which Young’s modulus was not conditioned by the in-plane orientation [33].

The titanium samples also had brittle fractures with small plastic deformation after their yield point was determined. The average value for the modulus of elasticity was around 100 GPa, a smaller value than the bulk one of 115 GPa [86]. Due to the fluctuation of the dimensions (especially thickness), there was a fluctuation in modulus values. In general, many research papers report a significant discrepancy between mechanical properties values and bulk materials [87,88,89]. The brittle fractures were also reported by Ogawa et al. for sputtered titanium films [90] but together with tiny maximum elongations in tensile tests. The deposition conditions may have determined this discrepancy in fracture behavior [91].

Tall et al. [92] used typical atomic force microscopy (AFM) to mechanically investigate different Ni–Ti films (samples 1, 2, 3 with film thickness 1.3–1.5 μm). They reported a Young’s modulus of 91.0 GPa for the first sample, which indicates that the material is austenitic, and a value for hardness of 3.2 GPa. For the third sample, the value for the Young’s modulus was 63.2 GPa in accordance with martensitic Ni–Ti after deformation induced by stress, and the hardness was 2 GPa. For the second sample, the deformed volume was partially transformed, and therefore, the value for Young’s modulus was 73.4 GPa while the hardness was 4.3 GPa. Hence, the experimentally determined Young’s moduli can be used to obtain a qualitative characterization of the magnitude of stress-induced state transformation in tiny volumes of Ni–Ti films.

The research presented in [60] includes a 2.7-μm gold film that was indented at several depths between 20 and 2000 nm. The measurements were performed for temperatures between 20 and 130 °C. For the gold film, the elastic modulus decreases with the increase in temperature values. For low indentation depths (less than 100 nm), the measured values of the modulus seem to be the same for each test temperature. For example, the modulus values are around 90 GPa for an indentation depth of 100 nm when tests are performed at room temperature, and the values decrease to 69 GPa when tests are performed at 130 °C. The factors that influence the values of Young’s modulus are the composition, orientation of the film material and crystal structure. The influence of the heat treatment on the modulus is nonexistent, as long as it does not influence the indicated factors. For nanocrystalline Au and Cu thin films, the influence of the temperature is greater when examined in contrast to the bulk Au and Cu.

**Figure 19:**
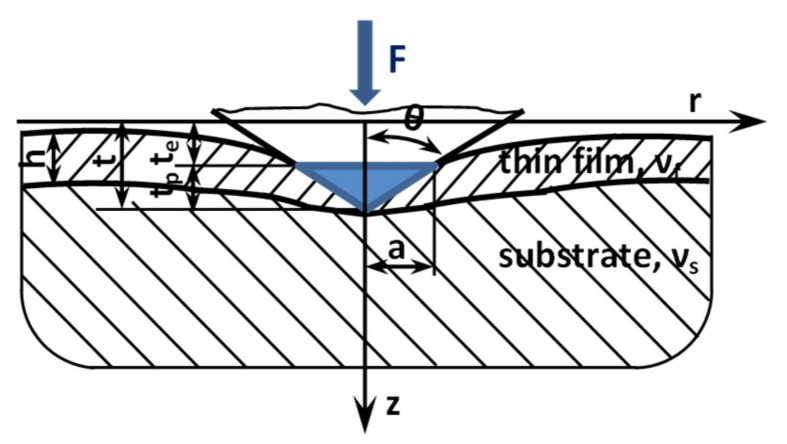
A Berkovich indenter penetrating a thin film/substrate medium.

Both the experimental investigations as well as the theoretical ones have shown that the values of Young’s modulus for a nanostructure are size-dependent. The research indicates that the modulus values are greater or smaller with respect to the size decrease, and some authors stated that this size influence is actually due to the so-called surface effects [93]. The unloading process of a rigid indenter penetrating a thin film on substrate is represented schematically in Figure 19. The thin film/substrate is considered a compliance contact of an uncracked film/substrate medium. According to Gao, Chiu, and Lee [94], who used the method of moduli-perturbation, the relation for the combined Young’s modulus E_eff_ of the film/substrate aggregation is given by:

(3) $$E_{\mathrm{eff}}=E_{s}+\left(E_{f}-E_{s}\right)\cdot I_{0}$$

where I_0_ is a function of the ratio h/a between the thickness of the film, and the contact radius is expressed as:

(4) $$I_{0}=\frac{2}{\pi }\mathrm{arctg}\left(\frac{h}{a}\right)+\frac{1}{2\pi \left(1-\upsilon \right)}\left[\left(1-2\upsilon \right)\cdot \frac{h}{a}\cdot \ln\frac{1+{\left(\frac{h}{a}\right)}^{2}}{{\left(\frac{h}{a}\right)}^{2}}-\frac{{\left(\frac{h}{a}\right)}^{}}{1+{\left(\frac{h}{a}\right)}^{2}}\right]$$

Theoretically and as presented in Figure 19, the total indentation t can be expressed as the sum of the plastic depth t_p_ and the elastic depth t_e_. On the other hand, during the unloading process, t is fully revocable, while t_p_ is expected to be constant. Considering this aspect, the unloading phase can be considered as the elastic contact between the thin film/substrate material and a rigid. The contact area is equivalent to that of the projected surface of a penetration area of the indenter. Based on the unloading curve, the elastic characteristics can be determined, and parameter t_p_ may not be equal to the real plastic penetration but rather corresponds to the depth of the contact region when unloading begins. According to [53,95], the effective contact compliance can be defined as a linear expression between the substrate and the thin film corrected by the ratio coefficient I_0_ (h/a).

The film modulus E_f_ is found by analytical computation of the experimental data, therefore, solving an elastic contact problem for a coated surface.

(5) $$E_{f}=\frac{E_{eff}+E_{s}\left(I_{0}-1\right)}{I_{0}}$$

In [96], the data were computed for each sample with Equation (5) for a value of 15μN for the applied force, resulting in the value of E_f_. The results are presented in Table 1.

Other authors have adopted a different method to measure the values of the elastic modulus for soft matter using the bimodal amplitude modulated-frequency modulated AFM technique. By using this novel technique, the topography and stiffness mappings of the analyzed samples can be obtained simultaneously. By assuming that there is a perfect Hertz contact between the tip and the sample, the equivalent elastic modulus can be determined [97,98].

The “plasticity index”, based on which the plasticity of a surface is determined, can be expressed as a function of the contact ratio of H to E. The main advantage of determining it comes when trying to avoid the wear [99,100]. The ratio H/E is a physical parameter and has been shown by a number of authors to be a more suitable parameter for predicting wear resistance than hardness alone. This ratio H/E actually indicates the film durability, and it is in correlation with the elastic strain and resilience of a material [99,101]. Since there are cases when tribological films are required to allow substrate deformation to an extent, it is crucial to have information on the failure capability.

The standard ISO 14577-4 recommends a linear extrapolation of modulus measurements from different depths to give a film-only value at zero indentation depth. This extrapolation procedure has been performed on the load–partial unload data to obtain film-only elastic modulus measurements. For thin-film systems with large differences in the elastic modulus between the film and substrate, this extrapolation procedure is essential to obtain true film-only modulus measurements.

**Figure 20:**
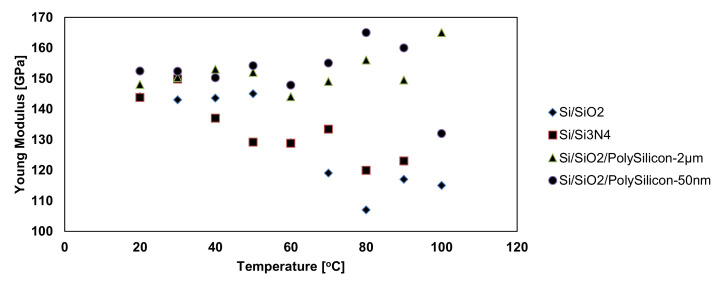
The Young modulus with respect to temperature for Si/SiO_2_, Si/Si_3_N_4_, Si/SiO_2_/PolySi—2 μm and Si/SiO_2_/PolySi—50 nm.

**Figure 21:**
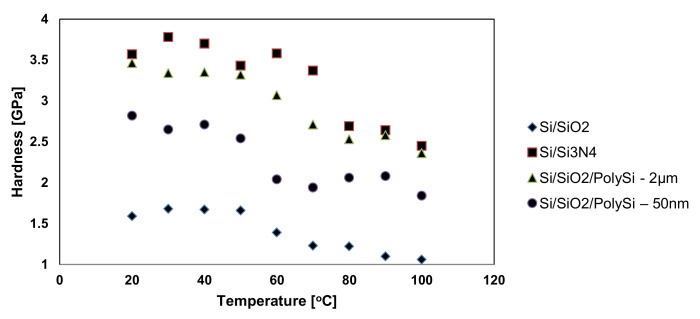
The hardness with respect to temperature for Si/SiO_2_, Si/Si_3_N_4_, Si/SiO_2_/PolySi—2 μm and Si/SiO_2_/PolySi—50 nm.

Birleanu et al. [96,102] determined the hardness of the analyzed thin films, incorporating the experimental data in the analytical model of Puch–Cabrera. This model takes into consideration the influence of the indentation size according to Meyer’s law. The used model is formulated based on a volume law of mixtures as follows:

(6) $$H_{\mathrm{eff}}=H_{s}+\left(H_{f}-H_{s}\right)\cdot \exp\left(-k{\left(\frac{t}{h}\right)}^{n}\right)$$

where the hardness rate of change when the boundary of the elastic behavior is crossed is given by the parameters k and n. Their values can be estimated by repetitive fitting of the experimental results. Usually, n is between 0.4 and 0.9, while k is between 1.9 and 4.9. On the other hand, the experimental results [12,103] show that both Young’s modulus (Figure 20) and hardness (Figure 21) become slightly smaller as temperature increases.

Woei S. Lee et al. [104] focused their investigations on the nanomechanical properties of an as-deposited Au/Cr/Si thin film and performed indentation tests at a maximum depth of 1500 nm. After indentation, the annealed thin-film specimens were thoroughly analyzed. The experimental results indicated that the unloading curve is characterized by a unique pop-out feature. The values obtained for the hardness at the maximum indentation depth was of 2.7 GPa and for Young’s modulus was of 110 GPa.

Other researchers investigated several polycrystalline thin films of copper of different thicknesses deposited on silicon substrates. At constant indentation loads, it was observed that the bursts of dislocation nucleation are delimited by an elastic deformation response proving Kick’s law for the sharp Berkovich indenter, a response that can be approximated by the thin film’s elastic properties [73].

## 5. Thin Film Nanotribological Properties

### 5.1. Friction Coefficient (CoF)

Mainly in the last two decades, there have been more experimental studies focused on examining the frictional parameters at atomic scales. It is a well-acknowledged fact that the frictional performance cannot be characterized only by considering the tribological system surface roughness, normal load, material type and speed. There are other factors to take into account, including sliding history, humidity and temperature. The condition of the surface peripheral layer is a factor whose impact on the tribological performance is increased at the nanoscale [105].

Another important aspect to consider when analyzing the tribological system characteristics is the RMS surface roughness, even if it does not provide any data on asperities frequencies, sizes and slopes [99,106]. Some studies show that CoF increases with respect to the increase in roughness values for carbon films [99,106,107]. Other investigations on the influence of surface roughness on friction phenomenon indicate large values of the CoF when the surfaces are very smooth or very rough. Many questions in MEMS have been encountered about wear and friction. In many MEMS sensors, these two phenomena are minimal; on the other hand, failure could be caused by friction and wear for most actuators.

The friction is described by Amontons’ laws at the macro scale, and according to them, it does not depend on the contact area and load. Experimental investigations prove that Amontons’ laws cannot be applied with success at the micro- and nanoscale. This happens due to the fact that the interdependency between tribological parameters and surface properties increases for miniaturized structures [108,109].

Lateral force microscopy (LFM) is a technique suitable to investigate the nanotribological behavior of the dynamic contact between different AFM probes and a hard layer film when the applied normal forces are between 5 and 100 nN. In order to determine the cantilever deflection (both lateral and vertical at the same time), the normal forces and the friction, this type of microscopy uses a position-sensitive photodetector (PSPD) [110].

**Figure 22:**
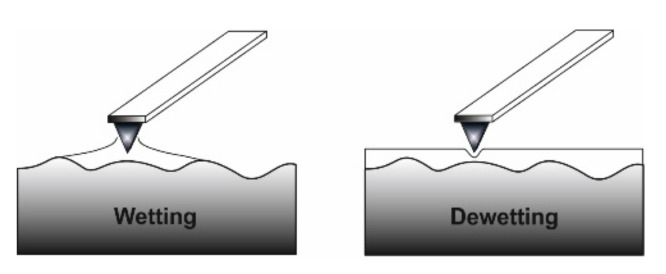
Schematic representation of wetting and dewetting of the AFM probe by the water layer.

The type of silicon probes used when testing with the AFM influences the layer films friction coefficients. Furthermore, the humidity existing in the environment affects the experimental results. This happens because a layer of water forms on the surface of the samples and on the probe. An attractive probe-sample force arises due to the meniscus that occurred between the water layer of the sample and the probe [111]. For forces lower than a critical load, it acts as a lubricant, while for a force greater than the critical load, the water layer is broken, and there is a frictional behavior that accompanies the interaction between the probe and the sample. According to G. Xie et al. [112] the contact between the probe and the sample is governed by the adhesive forces. In Figure 22, a schematic representation of the interaction of the AFM probe and surface sample is shown in the two possible cases: wetting (if the water layer becomes adhesive) and dewetting (if repellent-lubricant forces occur). Other researchers focused on the impact of the probe radius and relative humidity on the frictional behavior at the nanoscale. The tests encompassed a change in the radius in order to modify the contact surface in the experimental investigations. The obtained values indicated an increasing trend of the friction coefficient values with respect to the increase in contact surface and humidity.

There were researchers such as Bhushan and Kulkarni Kulkarni [113] and Tambe and Bhusan [114] who investigated the changes that occur in the frictional behavior when the scale is modified. Their results indicate a decrease in the friction coefficient with respect to the scale decrease. This phenomenon can be generated by the modification of the contact condition between the analyzed system components that lead to an alteration of the friction mechanism.

Ipaz L et al. [111] focused on investigating the nanotribological behavior of the AFM probe and hard multilayer film dynamic contact using LFM. The studies were performed at the nanoscale level, and normal forces of 5 to 100 nN were applied. They have shown that the AFM silicon probes used in the experimental investigations influence the CoFs for (Ti–Al/Ti–Al–N)n and (Ti–Cr/Ti–Cr–N)n multilayer films. The values for the surface roughness for the two types of investigated films vary between 2.8 and 3.5 nm and between 7.2 and 11.2 nm, respectively, when tests were performed with the sharpest diamond-coated tip. For these values, the variation of the CoFs is insignificant. The lubricant effect produced by the small water layer formed on the sample surface was observed for the experimental tests performed with a silicon tip coated by a Pt-Cr layer. This investigation allows the determination of the linear relation between the lateral force and the applied normal load when a critical threshold is reached for the force. The critical value of the normal force is reached when the water layer surface tension generates a repulsive force on the AFM probe. Diamond-coated tips become wet due to the water film created by the humidity in the environment, allowing the determination of the wanted linear relation even for smaller normal loads. These types of AFM tips also generate qualitative, accurate and detailed topographic images, and tests have shown that the lowest values for the CoF are obtained with these tips, namely 0.006–0.023 compared to 0.023–0.072 for the Pt–Cr-coated tip [111,115].

**Figure 23:**
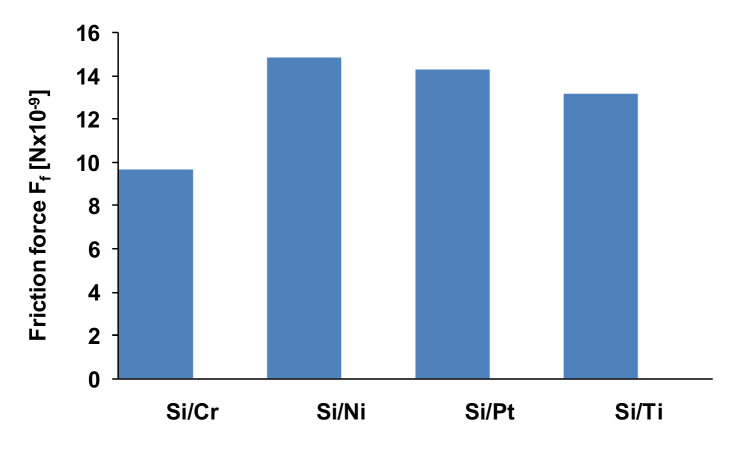
Friction forces for investigated samples.

**Figure 24:**
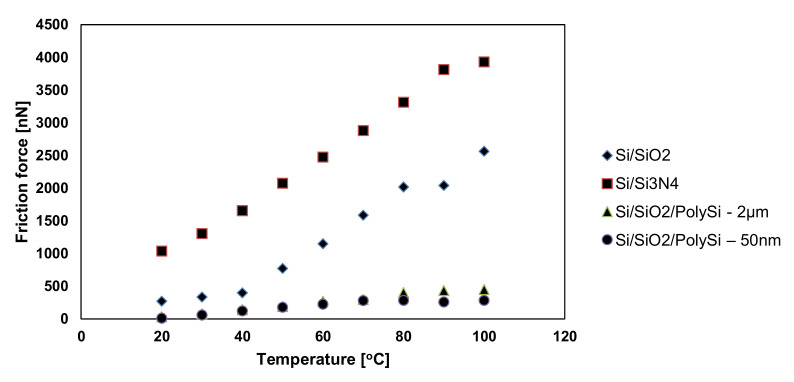
Friction force with respect to temperature for Si/SiO_2_; Si/Si_3_N_4_; Si/SiO_2_/PolySi—2 μm and Si/SiO_2_/PolySi—50 nm.

In [10,12,96,102,103,116,117], the procedure for obtaining the friction data consisted of measuring the cantilever lateral deflection while scanning the AFM probe back and-forth normal to the groove in contact with the sample at a setpoint of 10nN. After scanning the thin film with an AFM tip, the trace and retrace profiles were collected for each row of the topographic image and the chosen normal load was applied. Since the AFM silicon probes used in the experimental investigations influence the friction coefficients in the case of thin layer films, the friction force was determined considering the torsion beam theory (Figure 23). Based on the plots of the lateral force versus the applied normal load, the friction coefficient was determined. Figure 24 illustrates the influence on the friction force occurring between the AFM probe and the thin film for an environmental factor, namely the temperature.

As it can be seen for all investigated samples (Figure 24), the friction force has an increasing trend with respect to the temperature. However, the increase in the friction force values for Si/SiO_2_ and Si/Si_3_N_4_ occurs more rapidly, and it reaches the maximum values of 2600 and 4000 nN, respectively, while for the other samples, the increase is not so drastic and it reaches the maximum values of 450 and 300 nN, respectively.

The acquirements of a 3D-height map (morphologies of the thin films) of the scanned area for some samples are presented in Table 2.

Table 2 illustrates that the variations of the average roughness values (Ra) and ten-point mean height (Rz) values have the same trend as the variations of RMS roughness (Rq) values for all tested thin films. The statistical measurement of surface roughness is obtained with the histogram and a line profile along with an AFM image. For all other samples that have worked in the same way, the resulting data and processes are summarized in Table 2.

One source of random error in roughness measurements is the inevitable noise in images. As a result of image noise, assigned linewidths or edge positions, even for the same point on the sample, vary randomly from one measurement to the next.

A second source of error is the intrinsic randomness of the roughness itself. The roughness of a given line segment is one particular realization of a stochastic process. Even in the absence of any measurement error, the roughness of that segment will differ from that of another randomly chosen segment. If the size of this sampling uncertainty is larger than the allowed uncertainty of our roughness measurement, then it can be reduced by averaging the roughness variances from multiple segments. This average will be more representative of the average roughness than would be the roughness of a lone segment.

With increasing roughness, surfaces can be more hydrophobic [118], leading to reduced capillary force and adhesion. The effect of the surface on van der Waals forces has been studied by Meradudin and Mazur. Based on calculations, they found that surface roughness increases the magnitude of the van der Waals force over its value when the two surfaces are smooth [119]. The surface roughness as characterized by groove formation along grain boundaries is influenced by the manufacturing process. The AFM tip could follow the large topography variation faithfully and with no tip damage. The whole roughness study was completed with one AFM tip.

**Figure 25:**
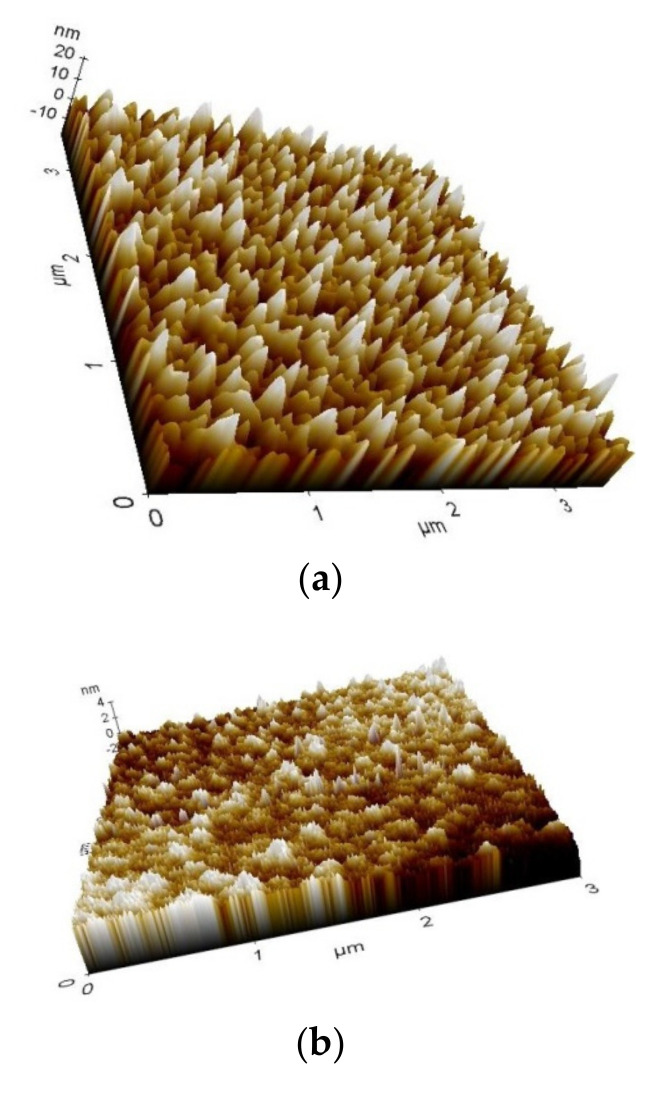

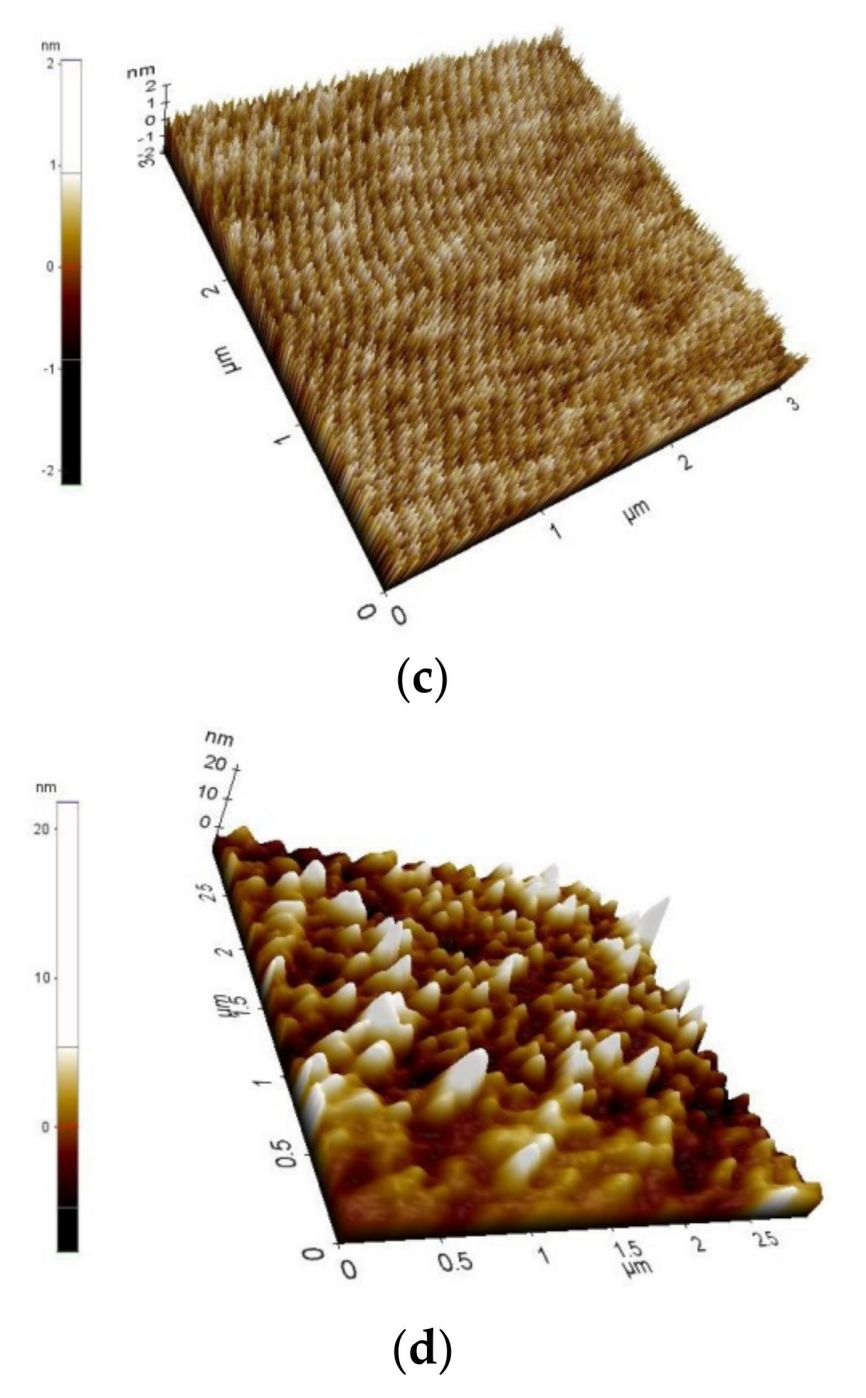
AFM-3D surface images of thin films samples. (**a**) TiO_2_/Si; (**b**) TiN/Si; (**c**) NbN/Si; (**d**) Au/Cr/SiO_2_/Si.

The acquirements of a 3D-height map (morphologies of the thin films) of the scanned area for some thin films are shown in Figure 25.

**Figure 26:**
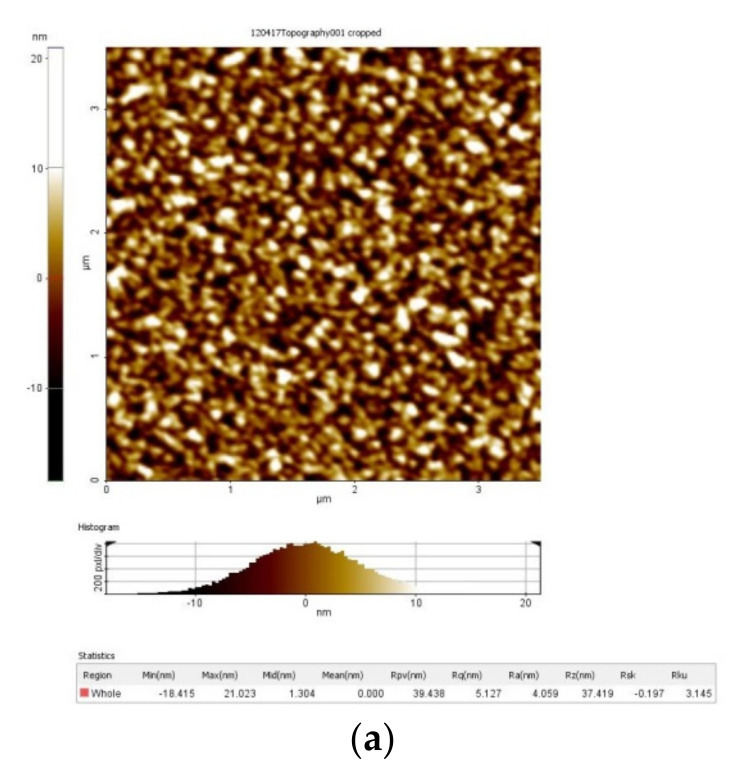

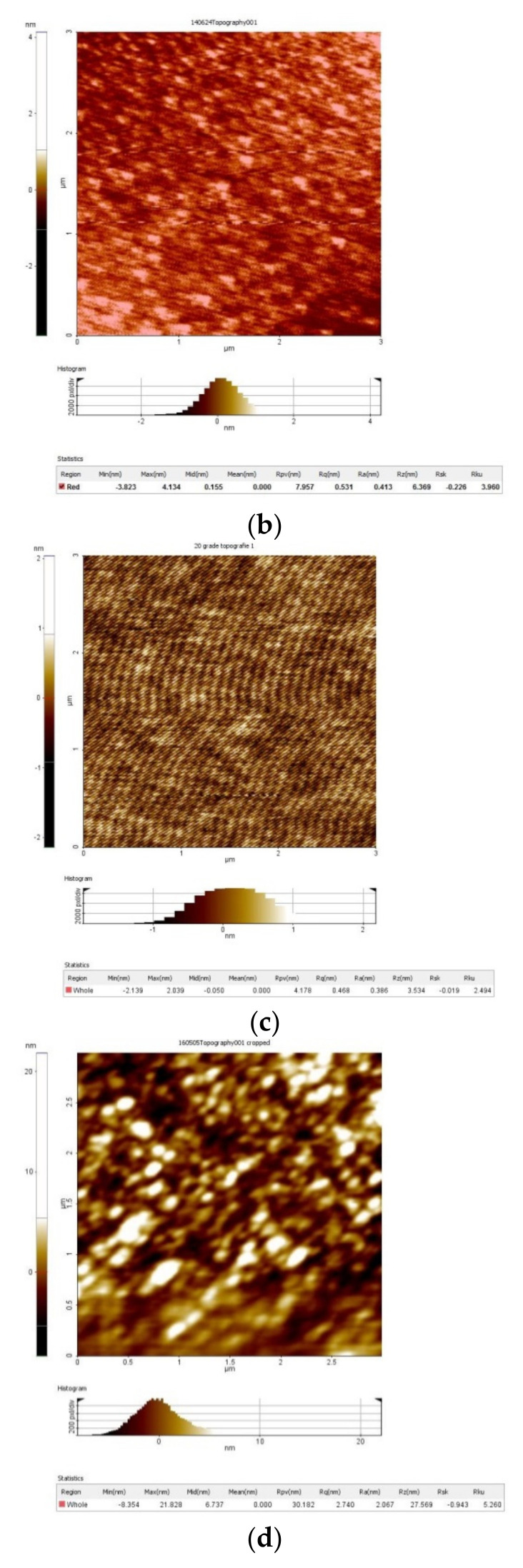
AFM-2D surface images of thin films samples (roughness parameters) (**a**) TiO_2_/Si; (**b**) TiN/Si; (**c**) NbN/Si; (**d**) Au/Cr/SiO_2_/Si.

The roughness can be characterized by several parameters and functions (such as height, wavelength, spacing and hybrid). It is useful for detecting general variations in overall profile height characteristics and for monitoring an established manufacturing process. Figure 26 represents acquirements of AFM images of surface morphology and roughness for different thin-film samples.

In several previous studies, while studying surface friction, surface roughness was neglected, and a smooth surface was assumed. It was much later that the importance of surface roughness in friction was realized. A prior study proved that surface texture and surface roughness affect frictional behavior during sliding. An extensive amount of research focuses on the understanding of the effect of surface roughness on nanofriction. Prior studies have shown that frictional force, between two laser-fabricated surfaces of nanoscale dimension with periodic grooves, is dependent on texture orientation.

The roughness is a very significant parameter for various applications. The characterization of materials through its roughness allows one to obtain information on the efficiency of materials in various application areas.

### 5.2. Adhesion

In addition to friction, adhesion is strongly impacted by the presence of even a tiny amount of water surfaces in contact. Wear, and ultimately failure after fewer cycles than estimated, is the consequence of friction, while permanent binding is the consequence of a strong enough adhesion. Methods for treating the surfaces such as using self-assembled monolayers (SAMs) are useful in lowering the hydrophilicity and thus the effect of the friction and adhesion [120].

The van der Waals attraction between molecules in proximity influences the strength of the interchain interactions and therefore the chain length, ultimately influencing the quality of a deposited monolayer. However, an aspect still to be investigated is what causes the friction decrease with respect to the increase in chain length.

The influence of the adhesion forces even in the case of contact with a rough surface is to be considered when computing the total surface load because the forces acting in MEMS devices are generally quite small [42]. Thus, the elastic contact model must incorporate the adhesion effect and its correlation to the roughness of the investigated surface.

**Figure 27:**
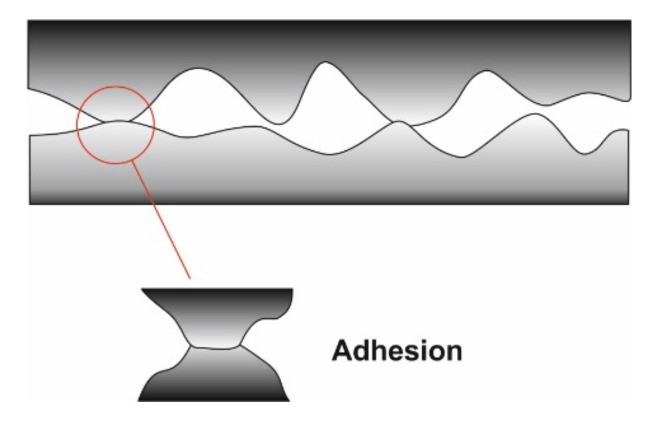
Schematic of adhesion model proposed by Bowden and Tabor.

The adhesion model illustrated in Figure 27 was proposed in 1954 by Bowden and Tabor and is found in most mechanisms of friction. Adhesion and contact pressure determined the formation of small junctions of asperities. The real contact surface is given by the combined surface of all the junctions.

(7) $$F_{f}=\tau \cdot A_{r};\;A_{r}=\frac{L}{p_{m}}$$

where: F_f_—frictional force; τ—the junction shear strength, A_r_—the real contact surface; L—the load applied externally and p_m_—the average value for the real contact pressure. The asperity summit level is dispersed, and the contact surface is proportional to the externally applied load for real surfaces with many points in contact.

The expression for the friction force is: $F_{f}=\mu_{c}\cdot L$ where $\mu_{c}=\frac{\tau }{p_{c}}$ and p_c_ is the average value for the real elastic contact pressure. The computed friction is increased when there is adhesion between the AFM probe and the thin film, and it is decreased by the thin-film tribochemical reactions that determine a film restructuring [121]. However, there is not enough data available to link adhesion and friction enough to estimate the relation that connects the reduction in the two studied parameters per unit [122]. Maneesh Mishra et al. have recently developed a mathematical model for the connection between the interface adhesion forces and the plastic deformation occurring at the subsurface, but only for single-asperity sliding contacts [122].

#### 5.2.1. Single Asperity Contact

Hertz’s equations describe the relation between the factors that intervene in the elastic deformation model of a single spherical asperity on a flat surface [123,124].

(8) $$a^{2}=R\cdot z$$

(9) $$P=\frac{a^{3}}{R\cdot D}=\frac{R^{1/2}\cdot z^{3/2}}{D}$$

where a is the contact radius, z is the indentation, P is the compressing force pressing the asperity and D is the composite compliance of the two materials that come in contact, given by:

(10) $$D=\frac{3}{4}\left(\frac{1-\nu_{1}^{2}}{E_{1}}+\frac{1-\nu_{2}^{2}}{E_{2}}\right)$$

where v_1,2_ and E_1,2_ are the Poisson ratios and, respectively, the Young’s moduli of the two surfaces that come in contact. Other models have been developed in order to encompass the adhesion effect. One of the simplest and most used ones is the DMT model, which is based on Hertz’s equations for the elastic deformation of an asperity, but it includes the adhesion forces acting between the asperity and the opposite surface. The total load is obtained by adding the adhesion forces and the expressions become:

(11) $$a^{2}=R\cdot z$$

(12) $$P+2\pi \cdot \Delta \gamma \cdot R=\frac{a^{3}}{R\cdot D}=\frac{R^{1/2}\cdot z^{3/2}}{D}$$

with Δγ (J/m^2^) being the work of adhesion.

If the adhesion forces occurring along the perimeter of the contact area modify the deformation profile, then the JKR model should be used. Tabor’s elasticity parameter is an indicator of which model should be chosen between the DMT and the JKR model [125].

#### 5.2.2. Multi Asperity Contact: The Greenwood and Williamson Model

**Figure 28:**
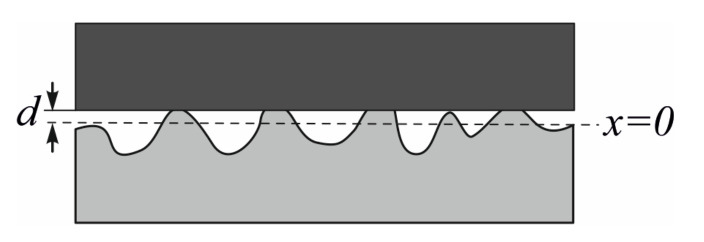
The separation d is defined as the level of the smooth plane with respect to the reference plane at x = 0.

A model for a multi asperity elastic contact has been developed by Greenwood and Williamson [126], who have considered the contact between a flat/smooth surface and a rough one (Figure 28). In this model, adhesion is not taken into consideration. The roughness of the surface is given by its asperities, which are considered to be spherical closer to their peaks, with a radius of $R={\left(\frac{1}{R_{1}}+\frac{1}{R_{2}}\right)}^{-1}$.

The preparation of the surface before the contact influences the distribution of the asperity height. Usually, the distribution of the peak levels is similar to a Gaussian distribution. Thus, the assumption of this distribution is made, with a standard deviation a and centered around the asperity height x = 0. The composite standard deviation of two rough surfaces is $\sigma^{2}=\sigma_{1}^{2}+\sigma_{2}^{2}$. After normalizing the asperity heights x and the separation d using $y=\frac{x}{\sigma }$ and $h=\frac{d}{\sigma }$ the expressions of the model developed by Greenwood and Williamson using a dimensionless representation [127] are:

(13) $$N^{*}=\frac{N}{\eta \cdot A_{a}}=F_{0}(h)$$

(14) $$A^{*}=\frac{A_{r}}{\eta \cdot R\cdot \sigma \cdot A_{a}}=\pi \cdot F_{1}(h)$$

(15) $$L^{*}=\frac{D\cdot L}{\eta \cdot A_{a}\cdot R^{1/2}\cdot \sigma^{3/2}}=F_{3/2}(h)$$

where N is the number of contact points, A_r_ is the real contact area and L is the load.

The normalized probability integrals for the assumed Gaussian distribution of the asperity peak levels are:

(16) $$F_{n}(h)=\frac{1}{\sqrt{2\pi }}\int_{h}^{\infty }{\left(y-h\right)}^{n}e^{\frac{y^{2}}{2}}dy$$

It is specific to the Gaussian distribution that the normalized contact pressure $\frac{L^{*}}{A^{*}}$ with respect to L* is approximately the same value. This happens because the load and the number of points in contact are approximately proportional. Therefore, there is almost no change in the value of the real contact surface per asperity and of the average real contact pressure. The average real elastic contact pressure $\frac{L}{A_{r}}$ can be determined from Equations (17) and (18):

(17) $$p_{c}=\frac{L}{A_{r}}=\sqrt{\frac{\sigma }{R}}\cdot \frac{F_{3/2}(h)}{D\cdot \pi \cdot F_{1}(h)}$$

Based on the DMT model, the statistical integrals can be computed and thus, the adhesion effect is included. The relations for A_r_, N, A*, N* are unchanged. However, Equation (15) becomes:

(18) $$L^{*}=\frac{D\cdot L}{\eta \cdot A_{a}\cdot R^{1/2}\cdot \sigma^{3/2}}=F_{3/2}(h)-\frac{2\pi }{\theta }\cdot F_{0}(h)$$

where θ is an adhesion parameter, given by Fuller and Tabor [119] as:

(19) $$\theta =\frac{\sigma^{3/2}\cdot R^{1/2}}{D\cdot \Delta \gamma \cdot R}$$

The adhesive load is highly influenced by any modification of the surface roughness because there is a proportionality relation between it and the number of asperities in contact. This can be proven using Equations (18) and (19) for computing a fraction of the asperities in contact with respect to the adhesion parameter. For example, if L* = 0 and the adhesion parameter θ is increased from 1 to 30, the number of asperities in contact drops from 1 to 0.001. Simultaneously, using the assumption that η⋅R⋅σ=0.1, the ratio $\frac{A_{r}}{A_{a}}$ representing the relative real contact area drops from 1 to 0.0001 [125].

The relation between the applied load and the real contact area is non-linear when the adhesive load is taken into consideration. However, there’s a linear equation that estimates the relation between L and F_f_, but solely for a small domain of L > 0:

(20) $$F_{f}=\mu_{e,a}\left(F_{a}^{0}+L\right)$$

where $F_{a}^{0}$ is the (apparent) zero load adhesion force that reflects the adhesion load influence and $\mu_{e,a}=\frac{dF_{f}}{dL}$ is the friction coefficient for elastic adhesive contact. A larger applied load determines an increase in the number of asperities in contact, contributing to a larger adhesive load, whose increase is reflected by the friction coefficient.

The model is based on elastic deformation in the contact points. The maximum pressure q_0_ in the Hertzian contact is given by:

(21) $$z=\frac{4}{9}\cdot \pi^{2}\cdot q_{0}^{2}\cdot D^{2}\cdot R$$

where z represents the indentation.

The plasticity index indicates, for a model based on the Gaussian distribution, the conditions for plastic or elastic contact. When the average real elastic contact pressure is greater than a fraction of the hardness: $p_{e}>0.24\cdot H$, serious plastic deformation occurs. This condition is true for adhesive as well as non-adhesive contact. The value of the average real contact pressure is close to the hardness H when $\sqrt{\frac{\sigma }{R}}$ is increased and p_e_ is larger than 0.24 H. If $p_{e}<0.24\cdot H$, then the friction coefficient is equal to $\frac{\tau }{p_{e}}$, thus it is much larger than $\mu_{p}=\frac{\tau }{H}$, which provides the lower limit. However, it is close to this limit when $p_{e}>0.24\cdot H$.

The surface morphology has a significant effect on adherence. The study presented in [116] focuses on determining the influence of RMS roughness on the adhesion using the AFM. The results encompassed in this study bring greater comprehension regarding the roughness influence on adhesion at the nanoscale level. It is known that at the nanoscale adhesion plays a critical role in MEMS applications. The influence of each molecule implicated in the contact between the surfaces is included in the expression for the total adhesion force:

(22) $$\frac{F}{R}=\frac{A}{6h_{c}^{2}}\left[\frac{1}{1+\frac{58.14\cdot R_{q}}{\lambda^{2}}}+\frac{1}{{\left(1+\frac{1.817\cdot R_{q}}{h_{c}}\right)}^{2}}\right]$$

where R is the radius of the tip (18 nm); R_q_ is the RMS of roughness; h_c_ is the minimum separating distance between the sample and the tip (0.3–0.4 nm), A is the Hamaker constant, λ is peak-to-peak distance and 2πωR represents the AFM system strength, where ω is the work of adhesion.

**Figure 29:**
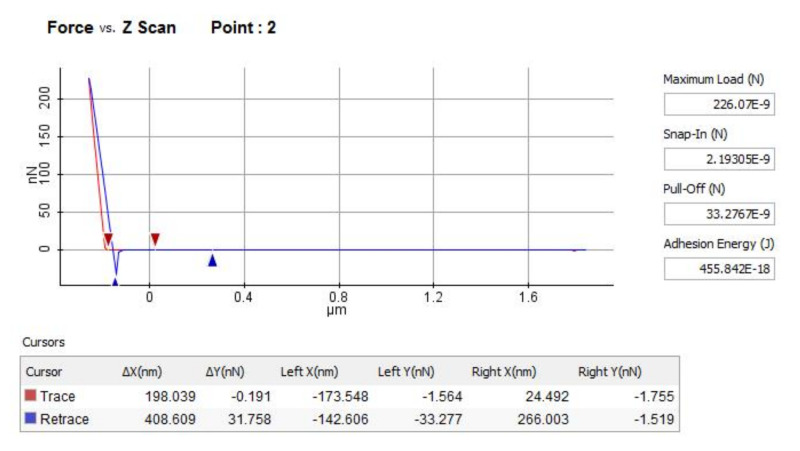
Adhesion forces for Si/Cr in 16th mapping area.

In the same study, using the technique of spectroscopy in point mode of the AFM, the adhesive force was measured at a frequency of 1Hz, and the nominal value of the spring constant of cantilever HQ—NSC 35/Hard/Al BS was k = 5.4 N/m. The adhesion force between AFM tip (Si_3_N_4_) and Si/Cr (also known as the pull-off force) is presented in Figure 29 [116]. The investigated area of 5 × 5 μm was divided into 16 (4 × 4) equally spaced areas, which were analyzed by the AFM technique collecting adhesion (pull off) forces.

After a statistical interpretation of the results, medium and dispersion values of pull off forces for different thin films are presented in Table 3 [116].

**Figure 30:**
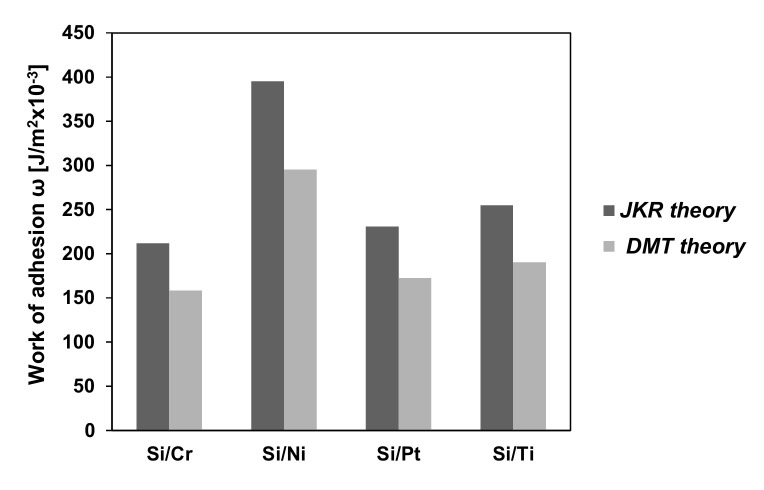
Graphical comparison of work of adhesion based on DMT and JKR theories.

Based on the experimental value [116] of the pull-off forces, the works of adhesion for the investigated samples are presented in Figure 30.

If the surface chemical composition of the tip or thin film is modified during the investigation, the pull-off forces occurring on the homogeneous surface can vary. Even if there is no observable geometrical change of the tip, it is possible that a chemical change occurred due to surface contaminations, material transfer or tribomechanical changes.

For the experimental tests, multiple pull-off measurements at different locations on the surfaces of the thin-film samples were taken (not so homogeneous surface), and it was observed that the variation in both the surface roughness and surface chemistry affect the interaction forces. If the surface topography and composition vary, the contact between the AFM tip and thin-film samples changes based on local slope, elasticity, curvature and adhesion. Furthermore, it was shown that the homogeneity of sample surfaces, as featured in adhesion maps, can be further decreased if the surface is contaminated. This was observed by exposing the thin-film samples around 100 min of air with constant humidity before the force curve measurements were taken.

**Figure 31:**
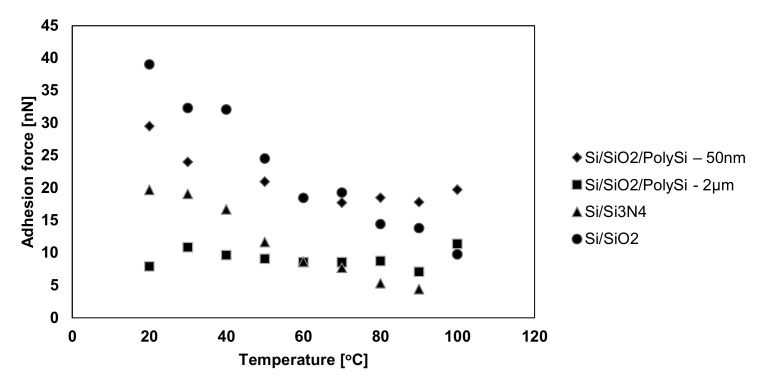
Adhesion force versus temperature for Si/SiO_2_, Si/Si_3_N_4_, Si/SiO_2_/PolySi—2μm, Si/SiO_2_/PolySi—50 nm.

In applications, ‘in-use adhesion’ issues occur due to the fact that the movable MEMS structures come in contact during operation. An initial solution to eliminate this problem was to manufacture micro dimples in the contact area or roughen the subtract surface in order to minimize the contact area. This solution minimized the adhesion by twenty times [16,128,129,130]. Thin films are not particularly useful if they do not stick. As with all microfabrication processes, ensuring the cleanliness of the surfaces will help here. Increasing surface roughness is also a useful technique, as it increases the effective surface area onto which the film can be deposited, and when creating a thin film of a metal on an oxide, including an oxide-forming element between the layers can also ensure good adhesion. It should also be pointed out that the changes in the measured adhesion forces can be due to heterogeneity in the contact area caused by the geometry of the tip and surface roughness, in addition to capillary effects that depend on the meniscus radius. A surface with nanometer roughness cannot be considered as a flat plane because the radius of the tip is also in the nanoscale. For example, when the tip approaches a peak region of the substrate, the measured adhesion is artificially lowered because the contact area between the tip and surface is small. To evaluate the influence of changing temperature on adhesion, the measurements were made on several sets of thin films [12,96,102,103,116]. When tests were performed between 20 and 35–40 °C, the results indicate only a small variation of the adhesive force values. However, that is not the case with the adhesion between the AFM tip and any of the thin-film samples at high temperatures due to factors such as reduction in surface tension of water and desorption of water. The results for the adhesion force between a Si_3_N_4_ AFM tip and several samples at temperatures between 20 and 100 °C are illustrated in Figure 31 [10,12,96,102,103,116].

At micro/nanoscales, friction is in a regime where the contribution from adhesion can outweigh that from the asperity deformation. Silicon shows a higher coefficient of friction value owing to its higher adhesion and higher contact area due to its higher interfacial energy. When compared to silicon, thin films/coatings have lower adhesion values, and hence, they exhibit lower values of friction coefficient.

## 6. Conclusions

In this review, we present an overview of the nano/micro-tribological properties of the adhesion, friction and wear durability of MEMS/NEMS materials, which includes various films/coatings. It is seen that the nano/micro-tribological properties of these materials are governed by several parameters, such as their physical structure, chemical composition, surface properties, including surface energy/wettability and resistance to interfacial shear, and mechanical properties, such as modulus of elasticity, hardness, adhesion and wear behavior, that influence the contact area.

Although most of these materials have improved tribological performance in terms of reducing surface forces and protecting silicon in MEMS/NEMS devices, it is important that they have long-term wear resistance. The wear durability of a tribological surface is a critical factor, probably even more so than the low friction, as it defines the useful life of the operation, and therefore, surface modifications for tribological applications in MEMS/NEMS devices should not only lead to reducing surface forces but should also effectively increase their wear durability.

In this review, an overview of AFM test methods and mechanical properties of thin film MEMS materials is provided. The lack of international norms on MEMS materials and the methods suitable for measuring their properties is a valid explanation for the differences in the results obtained for some common materials and reported in this study.

It is important to mention that even if MEMS is a technological field of quickly growing economic impact, a barrier to some extent in the development of reliable MEMS for special applications is the already mentioned absence of international norms that would provide a guideline to reliable evaluation with respect to material properties.

When designing a MEMS device for a certain application, the decision of choosing a structural material or coating is influenced by the specific application for which it is designed but also by the possibility of controlling the material deposition process and by the possibility of integrating it into the fabrication process of the device. For each real-world application, the most suitable coating material and/or method of deposition are still to be determined.

## Figures and Tables

**Table 1:** Analytical solution for thin-film elastic modulus and hardness.

Sample	Si/Cr	Si/Ni	Si/Pt	Si/Ti
E_f_ (GPa)	148.9566	120.0840	104.4746	109.5806
H_f_ (GPa)	9.002–9.108	9.127–9.298	8.97–9.18	9.204–9.322
H_f_/E_f_	0.055–0.061	0.076–0.077	0.086–0.088	0.084–0.085

**Table 2:** Roughness parameters of thin films (samples).

Samples	Roughness (nm)			
	R_a medium_	R_z medium_	R_q medium_	Max. Error (%)
Si/Cr	0.958	13.167	1.234	(±4.21%)
Si/Ni	0.238	2.848	0.303	(±3.23%)
Si/Ti	1.345	27.482	1.707	(±1.36%)
Si/Pt	2.097	21.931	2.659	(±3.19%)

**Table 3:** Medium and dispersion value of pull off forces.

Sample	Si/Cr	Si/Ni	Si/Pt	Si/Ti
Pull-off force medium (10^−9^ N)	17.966	33.524	19.585	21.6303
max. error (%)	(±4.438%)	(±3.858%)	(±2.758%)	(±3.878%)
σ_pull-off_	0.6524	0.3169	0.4437	0.839

## References

[1] Cao X., Jie Y., Wang N., Wang Z.L. (2016). Triboelectric Nanogenerators Driven Self-Powered Electrochemical Processes for Energy and Environmental Science. Adv. Energy Mater..

[2] Tambe N.S. (2005). Nanotribological investigations of materials, coatings and lubricants for nanotechnology applications at high sliding velocities. Ph.D. Thesis.

[3] Bhushan B. (2008). Physical and E. Sciences, Nanotribology, nanomechanics and nanomaterials characterization. Philos. Trans. A Math. Phys. Eng. Sci..

[4] Li X., Bhushan B., Takashima K., Baek C.-W., Kim Y.-K. (2003). Mechanical characterization of micro/nanoscale structures for MEMS/NEMS applications using nanoindentation techniques. Ultramicroscopy.

[5] Bhushan B. (2000). Modern Tribology Handbook, Two Volume Set.

[6] Maboudian R. (1997). Critical Review: Adhesion in surface micromechanical structures. J. Vac. Sci. Technol. B Microelectron. Nanometer Struct..

[7] Song Y., Nair R.P., Zou M., Wang Y. (2010). Adhesion and friction properties of micro/nano-engineered superhydrophobic/hydrophobic surfaces. Thin Solid Film..

[8] Nalwa H.S. (2001). Handbook of Thin Films, Five-Volume Set.

[9] Bharat B.J.B. (1999). Handbook of Micro/Nano Tribology.

[10] Voicu R.-C., Pustan M., Birleanu C., Baracu A., Müller R. (2015). Mechanical and tribological properties of thin films under changes of temperature conditions. Surf. Coat. Technol..

[11] Mehrpouya M., Bidsorkhi H.C. (2017). MEMS Applications of NiTi Based Shape Memory Alloys: A Review. Micro. Nanosyst..

[12] Birleanu C., Pustan M., Merie V., Müller R., Voicu R., Baracu A., Craciun S. (2016). Temperature effect on the mechanical properties of gold nano films with different thickness. IOP Conf. Ser. Mater. Sci. Eng..

[13] Hsu S.M. (2003). Nanolubrication: Concept and Design. Nanotribology.

[14] Kumar A., Yadav R., Janyani V., Prasad M. Structural study of aluminium nitride thin film grown by radio frequency sputtering technique. Proceedings of the International Conference on Computer, Communications and Electronics COMPTELIX.

[15] Gill J.J., Chang D.T., Momoda L.A., Carman G.P. (2001). Manufacturing issues of thin film NiTi microwrapper. Sens. Actuators A Phys..

[16] Fu Y., Du H., Huang W., Zhang S., Hu M. (2004). TiNi-based thin films in MEMS applications: A review. Sens. Actuators A Phys..

[17] Freund L.B., Suresh S. (2004). Thin Film Materials: Stress, Defect Formation And Surface Evolution.

[18] Cozma S., Vlǎdoiu R., Mandes A., Dinca V., Prodan G., Buršíková V. (2020). Characterization of Platinum-Based Thin Films Deposited by Thermionic Vacuum Arc (TVA) Method. Materials.

[19] Kumar P., Bansal D., Mehta K., Kumar A., Rangra K., Boolchandani D., Anuroop (2019). Optimization of Titanium Nitride Film for High Power RF MEMS Applications. J. Electron. Mater..

[20] TrueDyne MEMS Technology. https://www.truedyne.com/density-measurement-basics-part-3/?lang=en,Last.

[21] Sharma N., Hooda M., Sharma S.K. (2014). Synthesis and characterization of LPCVD polysilicon and silicon nitride thin films for MEMS applications. J. Mater..

[22] Guo Y.-B., Wang D.-G., Zhang S.-W. (2011). Adhesion and friction of nanoparticles/polyelectrolyte multilayer films by AFM and micro-tribometer. Tribol. Int..

[23] Zheng X., Zhou Y. (2005). Investigation of an anisotropic plate model to evaluate the interface adhesion of thin film with cross-sectional nanoindentation method. Compos. Sci. Technol..

[24] Tan C.W., Miao J. (2009). Optimization of sputtered Cr/Au thin film for diaphragm-based MEMS applications. Thin Solid Film..

[25] Harrison J., Schall J.D., Knippenberg M.T., Gao G., Mikulski P.T. (2008). Elucidating atomic-scale friction using molecular dynamics and specialized analysis techniques. J. Phys. Condens. Matter.

[26] Yoon E.-S., Singh R.A., Oh H.-J., Kong H. (2005). The effect of contact area on nano/micro-scale friction. Wear.

[27] Dedkov G. (1999). Theory of the noncontact friction forces between sliding nanoasperity and a surface. Wear.

[28] Ling Z., Qian L., Cheng G., Zhang Z., Sun D. (2013). Computer Simulation of Interactions between Copper and Aluminum Nano-films. Int. J. Sci. Eng. Comput. Technol..

[29] Iizuka T., Onoda A., Hoshide T. (2001). MD Simulation of Hardness Property of Al Thin Film Sputtered on Si Substrate and Its Related to Porosity. JSME Int. J. Ser. A.

[30] Xiang W., Zhao C., Liu K., Zhang G., Zhao K. (2015). Heteroepitaxial growth of TiN thin films on Si substrates for MEMS applications. J. Alloy. Compd..

[31] Gupta N., Dutta S., Pandey A., Vanjari S.R.K., Kaur D. (2020). Effect of growth and residual stress in AlN (0002) thin films on MEMS accelerometer design. J. Mater. Sci. Mater. Electron..

[32] Conde J.P., Gaspar J., Chu V. (2003). Low-temperature thin-film silicon MEMS. Thin Solid Film..

[33] Tsuchiya T., Tabata O. (2008). Reliability of MEMS.

[34] Stanimirović Z., Stanimirovic I., Takahata K. (2009). Mechanical Properties of MEMS Materials, Micro Electronic and Mechanical Systems.

[35] Bellitto V. (2012). Atomic Force Microscopy: Imaging, Measuring and Manipulating Surfaces at The Atomic Scale. Atomic Force Microscopy.

[36] Binnig G., Quate C.F., Gerber C.J. (1986). Atomic force microscope. Phys. Rev. Lett..

[37] Raczkowska J., Montenegro R., Budkowski A., Landfester K., Bernasik A., Rysz J., Czuba P. (2007). Structure Evolution in Layers of Polymer Blend Nanoparticles. Langmuir.

[38] Munz M.J. (2010). Force calibration in lateral force microscopy: A review of the experimental methods. J. Phys. D Appl. Phys..

[39] Bhushan B., Kasai T., Kulik G., Barbieri L., Hoffmann P. (2005). AFM study of perfluoroalkylsilane and alkylsilane self-assembled monolayers for anti-stiction in MEMS/NEMS. Ultramicroscopy.

[40] Papastavrou G., Akari S. (1999). Specific detection of interactions between uncharged surfaces in different solvents: High-resolution imaging by chemical force microscopy*. Nanotechnology.

[41] Kim S.-K., Jung M.-H., Kim H.-W., Woo S.-G., Lee H. (2005). Measurement of the strength of adhesion of resist patterns using an atomic force microscope. Nanotechnology.

[42] Bowden F.P., Tabor D. (1964). The Friction and Lubrication of Solids-Part II.

[43] Schuh C.A. (2006). Nanoindentation studies of materials. Mater. Today.

[44] Kathalingam A., Marimuthu K.P., Karuppasamy K., Chae Y.-S., Lee H., Park H.-C., Kim H.-S. (2019). Structural and Mechanical Characterization of Platinum Thin Films Prepared Electrochemically on ITO/Glass Substrate. Met. Mater. Int..

[45] Oliver C.W., Pharr G.M.J. (2004). Measurement of hardness and elastic modulus by instrumented indentation: Advances in understanding and refinements to methodology. J. Mater. Res..

[46] Ma D.D.D., Lee C.S., Au F.C.K., Tong S.Y., Lee S.T. (2003). Small-Diameter Silicon Nanowire Surfaces. Science.

[47] Fischer-Cripps A. (2004). A simple phenomenological approach to nanoindentation creep. Mater. Sci. Eng. A.

[48] Oyen M.L., Cook R.F. (2003). Load–displacement behavior during sharp indentation of viscous–elastic–plastic materials. J. Mater. Res..

[49] Storåkers B., Larsson P.-L. (1994). On Brinell and Boussinesq indentation of creeping solids. J. Mech. Phys. Solids.

[50] Swadener J.G., Taljat B., Pharr G. (2001). Measurement of residual stress by load and depth sensing indentation with spherical indenters. J. Mater. Res..

[51] Layton A.R., Adams T.M. (2010). Introductory MEMS: Fabrication and Applications.

[52] Cheng Y.-T., Cheng C. (2004). Reports, Scaling, dimensional analysis, and indentation measurements. Mater. Sci. Eng..

[53] Fischer-Cripps A.C. (2011). Contact Mechanics, in Nanoindentation.

[54] VanLandingham M.R. (2003). Review of instrumented indentation. J. Res. Natl. Inst. Stand. Technol..

[55] Lichinchi M., Lenardi C., Haupt J., Vitali R. (1998). Simulation of Berkovich nanoindentation experiments on thin films using finite element method. Thin Solid Film..

[56] Jung Y.-G., Lawn B.R., Martyniuk M., Huang H., Hu X.Z. (2004). Evaluation of elastic modulus and hardness of thin films by nanoindentation. J. Mater. Res..

[57] Chen S., Liu L., Wang T. (2005). Investigation of the mechanical properties of thin films by nanoindentation, considering the effects of thickness and different coating–substrate combinations. Surf. Coat. Technol..

[58] Reusch M., Cherneva S., Lu Y., Žukauskaitė A., Kirste L., Holc K., Datcheva M., Stoychev D., Lebedev V., Ambacher O. (2017). Microstructure and mechanical properties of stress-tailored piezoelectric AlN thin films for electro-acoustic devices. Appl. Surf. Sci..

[59] Chauhan S.S., Manhas S.K., Joglekar M. Fabrication of cantilever MEMs structure of C-axis grown AlN film for energy harvester application. Proceedings of the 2018 IEEE International Conference on Industrial Technology (ICIT).

[60] Volinsky A.A., Moody N.R., Gerberich W.W. (2004). Nanoindentation of Au and Pt/Cu thin films at elevated temperatures. J. Mater. Res..

[61] Verdyan A. Nano indentation inspection of the mechanical properties of gold nitride thin films. Proceedings of the 3rd International Conference on Cybernetics and Information Technologies, Systems, and Applicat/4th International Conference on Computing, Communications and Control Technologies.

[62] Soh M.T.K., Fischer-Cripps A.C., Savvides N. Elastic modulus of silicon nitride thin films from nanoindentation. Proceedings of the 16th National Congress of the Australian Institute of Physics, Congress Proceedings Handbook and Abstracts.

[63] Cao Y., Allameh S., Nankivil D., Sethiaraj S., Otiti T., Soboyejo W. (2006). Nanoindentation measurements of the mechanical properties of polycrystalline Au and Ag thin films on silicon substrates: Effects of grain size and film thickness. Mater. Sci. Eng. A.

[64] Sundararajan S., Bhushan B. (1998). Micro/nanotribological studies of polysilicon and SiC films for MEMS applications. Wear.

[65] Li X., Bhushan B. (1999). Micro/nanomechanical characterization of ceramic films for microdevices. Thin Solid Film..

[66] Luo J., Hu Y., Wen S. (2008). Physics and Chemistry of Micro-nanotribology.

[67] Sarkar L., Singh S.G., Vanjari S.R.K. (2021). Preparation and optimization of PVDF thin films for miniaturized sensor and actuator applications. Smart Mater. Struct..

[68] Koehler J.S. (1970). Attempt to Design a Strong Solid. Phys. Rev. B.

[69] Lehoczky S.L. (1978). Strength enhancement in thin-layered Al-Cu laminates. J. Appl. Phys..

[70] Ruud J.A., Jervis T.R., Spaepen F. (1994). Nanoindentation of Ag/Ni multilayered thin films. J. Appl. Phys..

[71] Choi Y., Van Vliet K.J., Li J., Suresh S. (2003). Size effects on the onset of plastic deformation during nanoindentation of thin films and patterned lines. J. Appl. Phys..

[72] Son D., Jeong J.-H., Kwon D. (2003). Film-thickness considerations in microcantilever-beam test in measuring mechanical properties of metal thin film. Thin Solid Film..

[73] Guo J.-G., Zhao Y.-P. (2005). The size-dependent elastic properties of nanofilms with surface effects. J. Appl. Phys..

[74] Krivtsov A.M., Morozov N.F. (2002). On mechanical characteristics of nanocrystals. Phys. Solid State.

[75] Yang F.J.J. (2004). Size-dependent effective modulus of elastic composite materials: Spherical nanocavities at dilute concentrations. J. Appl. Phys..

[76] Streitz H.F., Cammarata R.C., Sieradzki K. (1994). Surface-stress effects on elastic properties. I. Thin metal films. Phys. Rev. B.

[77] Zhang H., Sun C.T. (2004). Nanoplate Model for Platelike Nanomaterials. AIAA J..

[78] Workum V., de Pablo J. (2003). Local elastic constants in thin films of an fcc crystal. Phys. Rev..

[79] Nan C., Li X., Cai K., Tong J. (1998). Grain Size-dependent Elastic Moduli of Nanocrystals. J. Mater. Sci. Lett..

[80] Sharma P., Ganti S. (2003). On the grain-size-dependent elastic modulus of nanocrystalline materials with and without grain-boundary sliding. J. Mater. Res..

[81] Villain P., Beauchamp P., Badawi K., Goudeau P., Renault P.-O. (2004). Atomistic calculation of size effects on elastic coefficients in nanometre-sized tungsten layers and wires. Scr. Mater..

[82] Broughton J.Q., Meli C.A., Vashishta P., Kalia R.K. (1997). Direct atomistic simulation of quartz crystal oscillators: Bulk properties and nanoscale devices. Phys. Rev. B.

[83] Miller R., Shenoy V.B. (2000). Size-dependent elastic properties of nanosized structural elements. Nanotechnology.

[84] Fedorchenko A.I., Wang A.-B., Cheng H.H. (2009). Thickness dependence of nanofilm elastic modulus. Appl. Phys. Lett..

[85] Luo J. (2004). Young’s modulus of electroplated Ni thin film for MEMS applications. Mater. Lett..

[86] Chinmulgund M., Inturi R., Barnard J. (1995). Effect of Ar gas pressure on growth, structure, and mechanical properties of sputtered Ti, Al, TiAl, and Ti3Al films. Thin Solid Film..

[87] Keller R.-M., Baker S.P., Arzt E. (1998). Quantitative analysis of strengthening mechanisms in thin Cu films: Effects of film thickness, grain size, and passivation. J. Mater. Res..

[88] Arzt E.J. (1998). Size effects in materials due to microstructural and dimensional constraints: A comparative review. Acta Mater..

[89] Kraft O., Schwaiger R., Wellner P. (2001). Fatigue in thin films: Lifetime and damage formation. Mater. Sci. Eng. A.

[90] Ogawa H., Suzuki K., Kaneko S., Nakano Y., Ishikawa Y., Kitahara T. (1997). Tensile testing of microfabricated thin films. Microsyst. Technol..

[91] Tsuchiya T., Hirata M., Chiba N. (2005). Young’s modulus, fracture strain, and tensile strength of sputtered titanium thin films. Thin Solid Film..

[92] Tall P.D., Ndiaye S., Beye A.C., Zong Z., Soboyejo W.O., Lee H.-J., Ramirez A.G., Rajan K. (2007). Nanoindentation of Ni–Ti thin films. Mater. Manuf. Process..

[93] Hay J.L., O’Hern M.E., Oliver W.C. (1998). Tie Importance of Contact Radius for Substrate-Independent Property Measurement of Thin Films. MRS Proc..

[94] Huajian G., Cheng-Hsin C., Jin L. (1992). Elastic contact versus indentation modeling of multi-layered materials. Int. J. Solids Struct..

[95] Puchi-Cabrera E.J.S. (2002). Technology, A new model for the computation of the composite hardness of coated systems. Surf. Coat. Technol..

[96] Birleanu C., Pustan M. (2017). Size effect on stiffness and pull-off force of thermally actuated gold cantilevers. Rom. J. Tech. Sci. Appl. Mech..

[97] Sun Y., Hu Z., Zhao D., Zeng K. (2017). Mechanical Properties of Microcrystalline Metal–Organic Frameworks (MOFs) Measured by Bimodal Amplitude Modulated-Frequency Modulated Atomic Force Microscopy. ACS Appl. Mater. Interfaces.

[98] Garcia R., Proksch R. (2013). Nanomechanical mapping of soft matter by bimodal force microscopy. Eur. Polym. J..

[99] Hayward I., Singer I., Seitzman L. (1992). Effect of roughness on the friction of diamond on cvd diamond coatings. Wear.

[100] Leyland A., Matthews A. (2004). Design criteria for wear-resistant nanostructured and glassy-metal coatings. Surf. Coat. Technol..

[101] Zhao W., Pu J., Yu Q., Zeng Z., Wu X., Xue Q. (2013). A Novel strategy to enhance micro/nano-tribological properties of DLC film by combining micro-pattern and thin ionic liquids film. Colloids Surf. A Physicochem. Eng. Asp..

[102] Birleanu C., Pustan M. The effect of film thickness on the tribomechanical properties of the chrome-gold thin film. Proceedings of the 2016 Symposium on Design, Test, Integration and Packaging of MEMS/MOEMS (DTIP).

[103] Merie V.V., Bȋrleanu C., Pustan M.S., Negrea G., Pintea I.M. (2016). Analysis on temperature effect on the mechanical and tribological properties of titanium nitride thin films. IOP Conf. Ser. Mater. Sci. Eng..

[104] Lee W.-S., Liu T.-Y., Chen T.-H. (2009). Nanoindentation Behaviour and Microstructural Evolution of Au/Cr/Si Thin Films. Mater. Trans..

[105] Charitidis C.A., Koumoulos E.P., Dragatogiannis D.A. (2013). Nanotribological Behavior of Carbon Based Thin Films: Friction and Lubricity Mechanisms at the Nanoscale. Lubricants.

[106] Tomala A., Roy M., Franek F. (2010). Nanotribology of Mo–Se–C films. Philos. Mag..

[107] Wang J., Sottos N.R., Weaver R.L. (2004). Tensile and mixed-mode strength of a thin film-substrate interface under laser induced pulse loading. J. Mech. Phys. Solids.

[108] Arias D.F., Marulanda D.M., Baena A.M., Devia A. (2006). Determination of friction coefficient on ZrN and TiN using lateral force microscopy (LFM). Wear.

[109] Kumar D.D., Kumar N., Kalaiselvam S., Dash S., Jayavel R. (2015). Micro-tribo-mechanical properties of nanocrystalline TiN thin films for small scale device applications. Tribol. Int..

[110] Grill A., Patel V. (1993). Tribological properties of diamond-like carbon and related materials. Diam. Relat. Mater..

[111] Ipaz L. (2012). Nanofriction study using atomic force microscopy (AFM) of multilayers based in titanium, chromium and aluminum. Strength Mater..

[112] Xie G., Ding J., Zheng B., Xue W. (2009). Investigation of adhesive and frictional behavior of GeSbTe films with AFM/FFM. Tribol. Int..

[113] Bhushan B., Kulkarni A.V. (1996). Effect of normal load on microscale friction measurements. Thin Solid Films.

[114] Tambe N., Bhushan B. (2004). Scale dependence of micro/nano-friction and adhesion of MEMS/NEMS materials, coatings and lubricants. Nanotechnology.

[115] Kumar V., Singhal R. (2018). Impact of SHI on structural and mechanical behavior of intermetallic NiTi thin films. Phys. B Condens. Matter.

[116] Birleanu C., Merie V., Crisan H.G. (2019). Effect of film thickness on the tribo-mechanical properties of chrome-gold thin films. Proceedings of the Romanian Academy Series A-Mathematics Physics Technical Sciences Information. UTPRESS.

[117] Pustan M., Dudescu C., Birleanu C., Rymuza Z. (2013). Nanomechanical studies and materials characterization of metal/polymer bilayer MEMS cantilevers. Int. J. Mater. Res..

[118] Chau A., Régnier S., Delchambre A., Lambert P. (2010). Theoretical and Experimental Study of the Influence of AFM Tip Geometry and Orientation on Capillary Force. J. Adhes. Sci. Technol..

[119] Fuller K.N.G., Tabor D. (1975). The effect of surface roughness on the adhesion of elastic solids. Proc. R. Soc. Lond. Ser. A Math. Phys. Sci..

[120] Alley R.L. Surface roughness modification of interfacial contacts in polysilicon microstructures. Proceedings of the 7th International Conference Sensors and Actuators.

[121] Wong C.H. (2012). Friction at Nanoscale. J. Appl. Mech. Eng..

[122] Mishra M., Egberts P., Bennewitz R., Szlufarska I. (2012). Friction model for single-asperity elastic-plastic contacts. Phys. Rev. B.

[123] Timoshenko S., Goodier J.J.I. (1970). Theory of Elasticity.

[124] Derjaguin B., Muller V., Toporov Y. (1975). Effect of contact deformations on the adhesion of particles. J. Colloid Interface Sci..

[125] Tas N.C., Gui M. Elwenspoek. Static friction in elastic adhesive MEMS contacts, models and experiment. Proceedings of the IEEE Thirteenth Annual International Conference on Micro Electro Mechanical Systems (Cat. No. 00CH36308).

[126] Greenwood J.A., Williamson J.B.P. (1966). Contact of nominally flat surfaces. Proc. R. Soc. Lond. Ser. A Math. Phys. Sci..

[127] Gao G.T., Mikulski P.T., Harrison J.A. (2002). Molecular-Scale Tribology of Amorphous Carbon Coatings: Effects of Film Thickness, Adhesion, and Long-Range Interactions. J. Am. Chem. Soc..

[128] Grierson D.S. (2008). Nanotribological properties of nanostructured hard carbon thin films. Ph.D. Thesis.

[129] Suresh S., Nieh T.-G., Choi B. (1999). Nano-indentation of copper thin films on silicon substrates. Scr. Mater..

[130] Guo J.-G., Zhou L.-J., Zhao Y.-P. (2008). Size-dependent elastic modulus and fracture toughness of the nanofilm with surface effects. Surf. Rev. Lett..

